# Human Cyclophilin B forms part of a multi-protein complex during erythrocyte invasion by *Plasmodium falciparum*

**DOI:** 10.1038/s41467-017-01638-6

**Published:** 2017-11-16

**Authors:** Prem Prakash, Mohammad Zeeshan, Ekta Saini, Azhar Muneer, Sachin Khurana, Bishwanath Kumar Chourasia, Arunaditya Deshmukh, Inderjeet Kaur, Surabhi Dabral, Niharika Singh, Zille Anam, Ayushi Chaurasiya, Shikha Kaushik, Pradeep Dahiya, Md. Kalamuddin, Jitendra Kumar Thakur, Asif Mohmmed, Anand Ranganathan, Pawan Malhotra

**Affiliations:** 1 Malaria Biology Group, International Centre for Genetic Engineering and Biotechnology (ICGEB), Aruna Asaf Ali Marg, New Delhi, 110067 India; 2 Recombinant Gene Products Group, International Centre for Genetic Engineering and Biotechnology (ICGEB), Aruna Asaf Ali Marg, New Delhi, 110067 India; 3 Special Centre for Molecular Medicine, Jawaharlal Nehru University, Aruna Asaf Ali Marg, New Delhi, 110067 India; 4 Plant Mediator Lab, National Institute of Plant Genome Research, Aruna Asaf Ali Marg, New Delhi, 110067 India

## Abstract

Invasion of human erythrocytes by *Plasmodium falciparum* merozoites involves multiple interactions between host receptors and their merozoite ligands. Here we report human Cyclophilin B as a receptor for PfRhopH3 during merozoite invasion. Localization and binding studies show that Cyclophilin B is present on the erythrocytes and binds strongly to merozoites. We demonstrate that PfRhopH3 binds to the RBCs and their treatment with Cyclosporin A prevents merozoite invasion. We also show a multi-protein complex involving Cyclophilin B and Basigin, as well as PfRhopH3 and PfRh5 that aids the invasion. Furthermore, we report identification of a de novo peptide CDP3 that binds Cyclophilin B and blocks invasion by up to 80%. Collectively, our data provide evidence of compounded interactions between host receptors and merozoite surface proteins and paves the way for developing peptide and small-molecules that inhibit the protein−protein interactions, individually or in toto, leading to abrogation of the invasion process.

## Abstract (web summary)

Invasion of red blood cells by *Plasmodium falciparum* is a complex process and relies on several receptor-ligand interactions. Here, the authors show that human cyclophilin B binds *Plasmodium* surface protein PfRhopH3 and that interruption of this interaction reduces invasion by 80%.

## Introduction

Malaria remains a lethal disease in large parts of the world despite an effective drug treatment, increasingly as a result of the emergence of drug-resistant parasites. Its symptoms and pathology are a direct result of invasion of host erythrocytes by the *Plasmodium* merozoite^1, 2^, a complex process that requires coordinated interactions between host erythrocyte and parasite surface proteins, because of which it is an attractive target for vaccine and drug development. Although more than 50 merozoite surface antigens are expressed on *Plasmodium* merozoite surface, till date barely 7−10 possible interactions between them and their erythrocyte receptors have been well documented^3–5^. Of these, merozoite surface antigens from two main families: erythrocyte binding proteins (EBPs) and reticulocyte binding like protein (RH) have mainly been studied for their role(s) in erythrocyte invasion^6–8^. The parasite ligand PfRh5, for example, binds to Basigin, an interaction found to be essential for invasion by all tested *Plasmodium falciparum* strains^8–10^. Basigin has also been shown to be a druggable target for anti-malarial interventions as anti-Basigin antibodies effectively block erythrocyte invasion by different *Plasmodium* strains^11^.

Basigin has been referred to under a variety of names—CD147, OX-47 antigen and CE9 in rat; gp42 in mice; HT7, neurothelin and 5A11 antigen in chickens^12^. CD147 or Basigin, acts as an extracellular matrix metalloproteinase inducer that regulates a number of biological processes, such as spermatogenesis, lymphocyte responsiveness, and movement of monocarboxylate transporters^13^. These multiple activities of Basigin involve a number of interacting proteins^12^. Among several Basigin interacting proteins, Cyclophilins are an interesting class of proteins in terms of structural, functional, and medical implications^12, 14, 15^. Basigin functions as a signaling receptor for Cyclophilins A and B in a variety of immune cells and this interaction regulates inflammatory responses in a number of diseases, such as lung inflammation, cardiovascular disease, and rheumatoid arthritis^16^. Cyclophilins were discovered as host cell receptors for the potent immunosuppressive drug, Cyclosporin A^17^. Cyclophilins belong to the immunophilin class of proteins^18^ and some members of this family have been associated with parasitic diseases. Human malaria parasite *P. falciparum* encodes 13 immunophilins or immunophilin-like proteins; however, their exact functions are still unknown^19–21^.

In the present study, we use bacterial two-hybrid assay to identify human Cyclophilin B as a receptor for PfRhopH3 and show that CypB is present on the RBC surface and binds to the merozoites. Conversely, anti-RhopH3 antibodies inhibit the binding of Cyclophilin B to the merozoite surface. We demonstrate a multi-protein receptor ligand interaction involving human CypB and Basigin, and *Plasmodium* PfRh5 and PfRhopH3. Additionally, by screening a codon-shuffled library we identify a 98 (aa)-long de novo peptide that inhibits the interaction between CypB and PfRhopH3 by binding to CypB and blocks invasion of the RBC by the Merozoites. Together, these results indicate that a multi-protein complex is formed involving CypB and PfRhopH3 and small molecules or peptides against these interacting proteins can act as potential drug candidates.

## Results

### CypB is a receptor for PfRhopH3

**Fig. 1:**
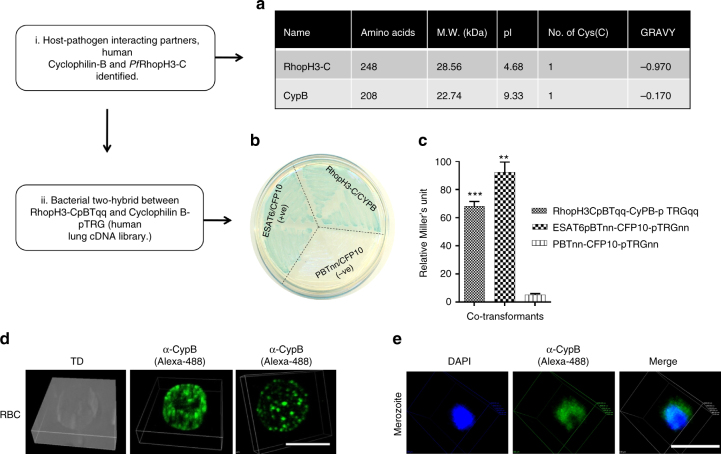
Cyclophilin B is a novel erythrocyte receptor for *P. falciparum* merozoite binding. **a** Parameters highlighting length, molecular weight (MW), Iso-electric point (pI), number of cysteines and grand average of hydropathicity (GRAVY) of the identified host-pathogen interacting protein partners Cyclophilin B (CypB) and PfRhopH3-C. **b** Bacterial two-hybrid assay between identified host-pathogen interacting partners. Streaks of the identified prey protein from the bacterial two-hybrid experiment between PfRhopH3-C and human lung cDNA library on X-gal indicator plate. All streaks are labeled to represent genes cloned in pBTnn and pTRGnn. CFP10-pTRGnn/empty pBTnn is the negative control; CFP10pTRGnn/ESAT6pBTnn is the positive control. **c** Liquid β-galactosidase assay to measure relative enzymatic activity of co-transformant pairs. Relative Miller’s units (M.U.) of RhopH3-CpBTqq/CYPBpTRGqq, CFP10pTRGnn/ESAT6pBTnn (positive control) and CFP10-pTRGnn/empty pBTnn (negative control) were plotted. The graph is the average of three independent assays with error bars representing the standard deviation; all values were tested for significance using a two-tailed unpaired Student’s *t*-test with Welch’s correction. **P < 0.01, ***P < 0.001. **d** Localization of CypB on the RBC surface. Human erythrocytes were incubated with primary anti-CypB monoclonal antibody (mouse) followed by secondary alexa-fluor 488 conjugated goat anti-mouse IgG antibody (1:200) and confocal microscopy. **e** Binding of CypB on the merozoite surface. Merozoites were incubated with 25 µM recombinant CypB for 2 h followed by incubation with primary anti-CypB monoclonal antibody (mouse). The Merozoites were stained with alexa-fluor 488 conjugated goat anti-mouse IgG antibody (1:200; green) against primary antibody followed by confocal microscopy. Scale bar = 5 µm

**Fig. 2:**
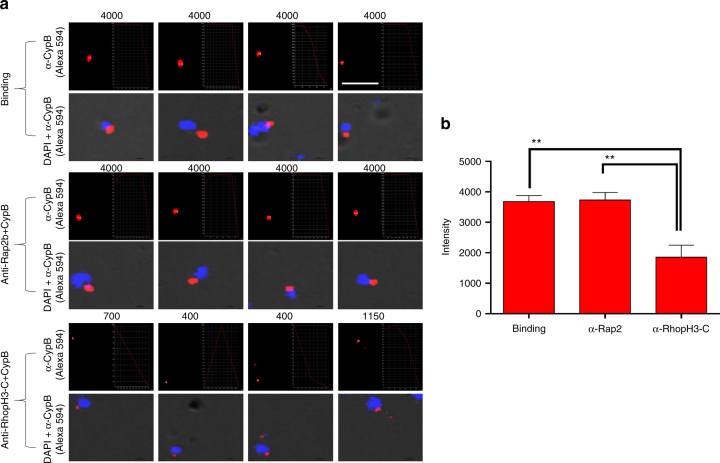
PfRhopH3 protein is a ligand for Cyclophilin B protein during merozoite binding. **a** Binding of Cyclophilin B on Merozoite surface. Binding shows binding of CypB on merozoite surface without treatment with any antibody. anti-Rap2b + CypB and anti-RhopH3-C + CypB show binding after treatment with anti-Rap2b and anti-RhopH3 antibody respectively. Intensity for each merozoite is mentioned at the top. Images with histograms represent only the intensity of the bound CypB protein. Anti-RhopH3-C antibody interferes with binding of CypB on merozoites, signifying that PfRhopH3 is a ligand for host Cyclophilin B. Scale bar = 5 µm. **b** Graph adapted from Supplementary Table 1 showing the average of binding intensity of CypB on 10 different merozoites, with error bars representing the standard deviation. All values were tested for significance using a two-tailed unpaired Student’s *t*-test. **P < 0.01

To look for novel host RBC and *Plasmodium* merozoite interactions, we used a bacterial two-hybrid^22, 23^ approach and screened a human tissue cDNA library against the PfRhopH3-C-terminal region (aa 617–865, Supplementary Fig 1). *Plasmodium* Rhoptry proteins, as it has been shown previously, act as important ligands for host receptors during the invasion process^24^. Specifically, PfRhopH3 has been shown to form a complex with the merozoite protein MSP1 and subsequently interact with erythrocyte Band 3 proteins to facilitate invasion^25, 26^. On the basis of blue−white selection, a putative colony positive for interaction was selected and the isolated DNA sequenced. Sequence analysis showed the host interacting partner of PfRhopH3-C as the full-length (aa 1–208) human Cyclophilin B (Fig. 1a, Supplementary Table 4). The plasmids harbored in the selected colony were segregated, confirmed by PCR, and used to co-transform competent R1 *Escherichia coli* cells to verify the interaction. Two-hybrid plasmids expressing the *Mycobacterium tuberculosis* proteins ESAT6 and CFP10, and whose interaction has been well-documented previously^22^ acted as the positive control. These interactions are shown in Fig. 1b, c. As Cyclophilin B possesses endoplasmic reticulum (ER) directed signal sequence and is secreted out in chondrocytes cells^27, 28^, we analyzed its erythrocyte surface expression. As shown in Fig. 1d, immunolocalization analysis using anti-Cyclophilin B antibody stained the human RBC well, thereby demonstrating the expression of Cyclophilin B on the RBC surface. To investigate whether Cyclophilin B acts as a receptor for the binding of the merozoite to the RBC, we assessed the binding of Cyclophilin B protein to free *P. falciparum* merozoites. We have previously performed similar studies to show ICAM-4 binding to merozoites^22^. Cyclophilin B showed significant binding on the merozoite surface (Fig. 1e), and co-localized with PfRhopH3 (Supplementary Fig. 2e), thus suggesting it to be a receptor for merozoite binding of human RBCs. Further, we developed an intensity measurement-based Cyclophilin B-merozoite binding assay. Briefly, *P. falciparum* free merozoites were incubated with 25 µM of Cyclophilin B protein. Intensity of Cyclophilin B binding on *P. falciparum* merozoite surface was quantified on Nikon fluorescent confocal microscopy using NIS element. Figure 2a shows Cyclophilin B binding intensity on four merozoites. For intensity measurements, the mean binding intensity for ten merozoites was calculated (Fig. 2a, b, Supplementary Table 1 and Supplementary Fig. 4).

### PfRhopH3 acts as a ligand of CypB for merozoite binding

**Fig. 3:**
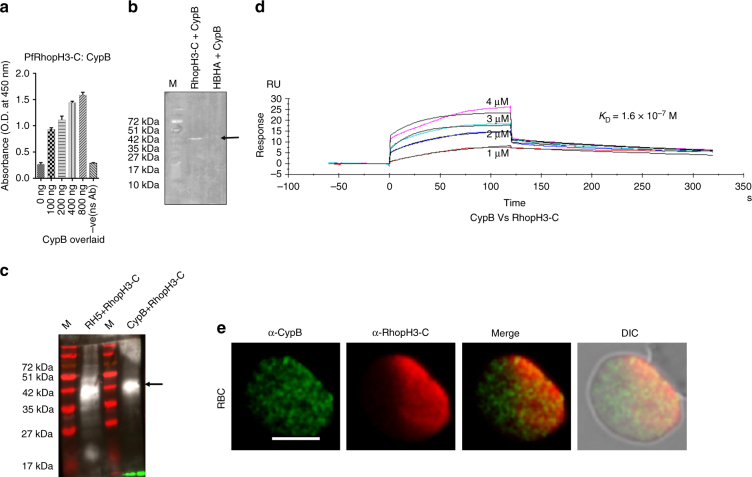
Protein−protein interaction assays confirm the interaction between Cyclophilin B and PfRhopH3-C. **a** ELISA-based assay confirming the interaction between CypB and PfRhopH3-C. 200 ng of PfRhopH3-C protein was coated on ELISA plates. CypB was overlaid on subset of wells in increasing concentrations as shown, and detected by anti-CypB (rabbit polyclonal) antibody followed by HRP conjugated secondary anti-rabbit antibody. A non-specific antibody ((nsAb) anti-CFP10 rabbit polyclonal) was used as the antibody control. Negative control for this ELISA experiment is shown in Supplementary Fig. 5d. Each bar represents the mean ± S.D. for triplicate experiment. **b** Far-western analysis. PfRhopH3-C was run on SDS-PAGE and transferred onto PVDF membrane and following denaturation and renaturation, incubated with CypB protein. CypB protein (indicated by arrow) was detected by anti-CypB (rabbit polyclonal) antibody. Mycobacterial protein HBHA was used as the negative control protein. **c** Co-immunoprecipitation of PfRhoph3-C with CypB and PfRh5. Antibody coupling resin bound with CypB and PfRh5 antibodies was incubated with CypB:PfRhopH3-C and PfRh5:PfRhopH3-C mixtures, respectively. Eluted samples were run on SDS-PAGE followed by western blotting. Detection of protein PfRhopH3-C (indicated by arrow) was carried out using anti-PfRhopH3 (rabbit polyclonal) antibody (1:5000). CFP10 was used as the negative control protein. **d** Surface plasmon resonance analysis of CypB and PfRhopH3-C interaction. The indicated concentrations of purified PfRhopH3-C were injected over immobilized CypB, and biophysical parameters were derived from a 1:1 binding model. RU, response units. *K*_D_ value for this interaction was 1.6 × 10^−7^ M. **e** Binding and co-localization of PfRHopH3-C on the RBC surface. Uninfected RBCs were incubated with 20 μM PfRhopH3-C and stained with anti-RhopH3-C antibody (rabbit) and anti-CypB antibody (mouse). Subsequently, secondary anti-rabbit alexa-flour 594 antibody and anti-mouse alexa-flour 488 antibody were used to detect the co-localization of the proteins. Scale bar = 5 µm

To confirm PfRhopH3 as a ligand involved in binding to Cyclophilin B on the RBC surface, we expressed and purified the C-terminal of PfRhopH3 protein (aa 617–865) in a heterologous *E. coli* expression system under denaturing condition (Supplementary Fig. 2a). Recombinant PfRhopH3-C protein was subsequently refolded by dialyzing it against a decreasing concentration of the denaturant. Antibodies were raised against the refolded PfRhopH3-C protein (Supplementary Methods). Supplementary Fig. 2b shows the differential migration of refolded PfRhopH3-C protein on SDS and Native PAGE under reducing and non-reducing conditions, suggesting proper folding of the protein. We next assessed the binding of recombinant PfRhopH3-C to the RBC surface. As shown in Supplementary Fig. 2d, PfRhopH3-C protein bound specifically to the RBCs and co-localized with human CypB on the RBC surface with a Pearson’s coefficient 0.62 (Fig. 3e). To determine whether PfRhopH3 is the ligand for Cyclophilin B, intensity measurement-based Cyclophilin B merozoite-binding assay was performed in the presence of anti-RhopH3 antibody. Briefly, free merozoites were incubated with anti-PfRhopH3 or anti-PfRAP2 antibodies where after washing, the treated merozoites were incubated with Cyclophilin B protein at a concentration of 25 µM. Cyclophilin B binding was assessed on the treated merozoites and compared with untreated merozoites. As shown in Fig. 2b, Supplementary Table 1, Supplementary Fig. 4, merozoites treated with anti-RAP2 antibody displayed similar binding intensity for Cyclophilin B as shown with the untreated control merozoites. However, more than 50% reduction in Cyclophilin B binding to merozoite surface was observed when the merozoite surface was treated with anti-RhopH3-C antibody, in comparison to merozoites treated with anti-RAP2 antibody or untreated cells (Fig. 2a, b, Supplementary Table 1, Supplementary Fig. 4). These results demonstrate that Cyclophilin B is the receptor for a merozoite surface localized Rhoptry protein PfRhopH3, which is known to be part of a large PfRhopH complex involved in erythrocyte binding^1, 29^. We further performed in vitro interaction studies between Cyclophilin B and PfRhopH3 proteins. Both, ELISA-based binding analysis and Far-western analysis showed a tight and specific interaction between Cyclophilin B and PfRhopH3 proteins (Fig. 3a, b). A reciprocal ELISA was also performed to further confirm the interaction between CypB and PfRhopH3-C (Supplementary Fig. 3c). Co-immunoprecipitation analysis further confirmed an interaction between Cyclophilin B and PfRhopH3 (Fig. 3c). To quantify the interaction between Cyclophilin B and PfRhopH3, surface plasmon resonance (SPR) analysis was performed. Cyclophilin B displayed a strong interaction with PfRhopH3 with an equilibrium dissociation constant *K*_D_ value of 1.6 × 10^−7^ M (Fig. 3d). Together, these results confirmed the involvement of a novel receptor-ligand (Cyclophilin B-PfRhopH3) interaction in the invasion of human erythrocytes by *P. falciparum* merozoites.

### CypB interacts with a critical merozoite receptor Basigin

**Fig. 4:**
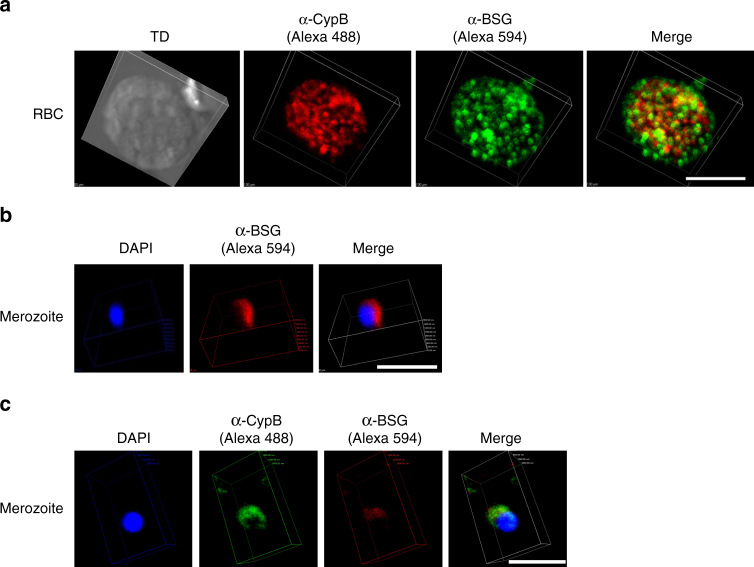
Cyclophilin B interacts with Basigin (CD147/BSG), a critical merozoite invasion receptor on RBCs. **a** Co-localization of CypB and BSG on the surface of the RBC. RBCs were fixed and incubated with anti-CypB (mouse monoclonal) and anti-BSG (rabbit polyclonal) antibodies. The cells were stained with fluorochrome-conjugated secondary antibodies against CypB (green) and BSG (red) followed by confocal microscopy. CypB and BSG co-localized on the RBC surface with a Pearson’s coefficient of 0.53. **b** Merozoites were incubated with 25 µM of BSG protein for 2 h and detection was carried out using anti-CD147 antibody (rabbit polyclonal) and secondary fluorochrome-conjugated (red), followed by confocal microscopy. **c** CypB and BSG bind together on the merozoite surface. Merozoites were incubated with CypB and BSG proteins (25 µM each) for 2 h and binding was detected using anti-CypB (mouse monoclonal) and anti-BSG (rabbit polyclonal) antibodies followed by secondary antibodies and confocal microscopy. Supplementary Fig. 5 illustrates in vitro interaction assays between CypB and BSG. Scale bar = 5 µm

Basigin is expressed in many cell-types such as hematopoietic cells including the human erythrocytes^13, 16^. Basigin has been shown to be a signaling receptor for extracellular Cyclophilins A and B, through which Cyclophilins mediate chemotactic activities^12, 30^. Cyclophilin A-Basigin interaction also regulates inflammatory responses in a number of diseases and is a target for new anti-inflammatory therapeutics^12^. To investigate whether Cyclophilin B and Basigin are co-expressed at the same time on human RBC surface, co-immunolocalization studies using anti-Cyclophilin B and anti-Basigin antibodies were performed. Both these antibodies stained human erythrocytes, thereby showing the existence and expression of both these receptors on RBC surface simultaneously (Fig. 4a). We next performed co-binding studies with these proteins on the merozoite surface. Cyclophilin B as well as Basigin bound to merozoites simultaneously (Fig. 4b, c), thereby indicating that these proteins bind to independent ligands on the merozoite surface that are, most likely, PfRhopH3 and PfRh5 respectively.

### A multi-protein complex involves Basigin-CypB-PfRhopH3-PfRh5

As mentioned earlier, Basigin has been reported to bind with *Plasmodium* merozoite protein PfRh5^8, 31^. A previous study has shown the co-localization of PfRh5 and PfRhopH3 on merozoite surface^32^ but their physical interaction has not yet been illustrated. Therefore, to determine whether Cyclophilin B specifically interacts with Basigin during *Plasmodium* merozoite invasion of RBCs and involved in complex formation, we carried-out protein−protein interaction analysis between Cyclophilin B and Basigin, between PfRh5 and PfRhopH3, and between Basigin and PfRh5. ELISA-based binding studies were performed as described earlier^22^. All of the above-mentioned interactions turned out to be positive and furthermore, robust, thereby indicating the existence of a multi-protein interaction between the two *P. falciparum* merozoite surface ligands and the two corresponding RBC surface proteins (Supplementary Fig. 5a–d). Further, co-localization of PfRhopH3 and PfRh5 was confirmed on the merozoite surface with a Pearson’s coefficient value of 0.62 (Supplementary Fig. 3b). Interactions between Cyclophilin B and Basigin, PfRh5 and PfRhopH3 were also quantified by SPR analysis (Supplementary Fig. 5g, h). Both these interactions were strong with *K*_D_ values of ~4.3 × 10^−7^ M and 1.8 × 10^−8^ M respectively. A previous study^8^ has reported a strong interaction between Basigin and PfRh5 with a *K*_D_ value of 1.12 × 10^−6^ M. We confirmed these interactions by Far-western blot and co-immunoprecipitation followed by Western blot analysis (Fig. 3b, c, Supplementary Fig. 5e, f). Together, these results strongly advocate an association between Cyclophilin B and Basigin on the RBC surface and between PfRh5 and PfRhopH3 on the merozoite surface. Based on these interaction analyses, we built a model shown in Supplementary Fig. 13.

### A de novo peptide CDP3 binds CypB and blocks invasion

**Fig. 5:**
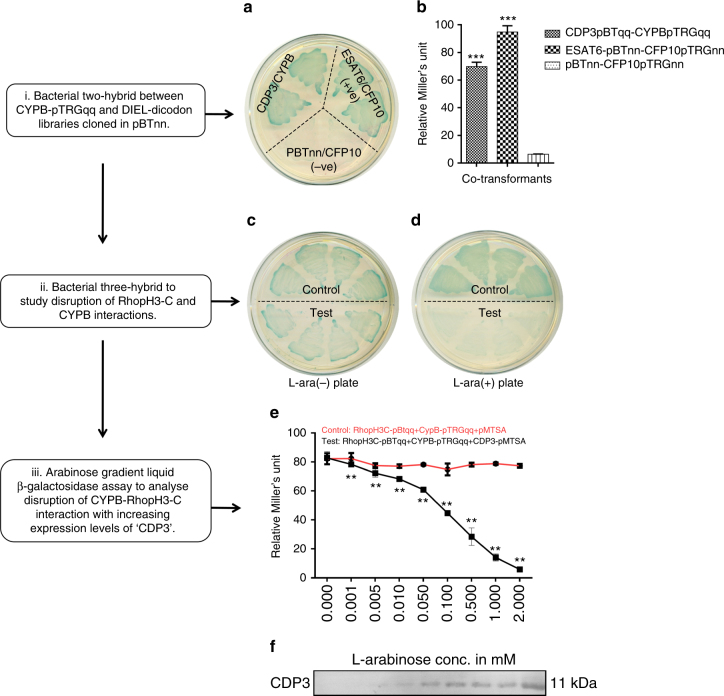
Identification of a de novo peptide that binds Cyclophilin B. **a** Bacterial two-hybrid assay to identify de novo interacting partner against CYPB from dicodon library. Streaks of bacterial two-hybrid experiments between DIEL-pBTnn and CYPBpTRGqq on X-gal indicator plate. All streaks are labeled to represent genes cloned in pBTnn and pTRGnn. CFP10pTRGnn/Emptyp BTnn and CFP10pTRGnn/ESAT6pBTnn signify the negative and positive controls, respectively. **b** Liquid β-galactosidase assay to estimate the relative enzymatic activity in Miller’s unit (M.U.) of the interaction between identified de novo dicodon library interacting partner (CDP3) and CYPB. The graph is the average of three independent assays with error bars representing the standard deviation. All values were tested for significance using a two-tailed unpaired Student’s *t*-test with Welch’s correction. ***P < 0.001. **c**, **d** Bacterial three-hybrid assay to monitor the disruption of PfRhopH3-C/CypB interaction by CDP3 in vivo. X-Gal indicator plate without l-arabinose and X-gal indicator plate with l-arabinose. Test streaks: Triple co-transformants containing PfRhopH3CpBTqq, CYPBpTRGqq, and CDP3pMTSA; control streaks: triple co-transformants containing PfRhopH3-CpBTqq, CYPBpTRGqq, and empty pMTSA. **e** l-arabinose gradient liquid β-galactosidase assay. Relative β-galactosidase activity Miller’s unit (M.U.) of the triple co-transformants containing PfRhopH3-CpBTqq, CYPBpTRGqq and empty pMTSA (red line, control) and triple co-transformants containing PfRhopH3CpBTqq, CYPBpTRGqq, and CDP3pMTSA (black line, test), is plotted against a range of l-arabinose concentrations. The graph is the average of three independent assays and the standard deviation is represented as the error bars. Multiple unpaired *t*-tests to compare enzyme activity of each triple co-transformant across individual l-arabinose concentrations were used. Statistical significance was determined using the unpaired *t*-test with Welch’s correction. **P < 0.01 was considered significant. **f** Western blot of triple co-transformants R1 *E. coli* whole-cell lysates shown in **e**, in order to analyze the concomitant expression of CDP3 protein with increasing concentrations of l-arabinose

To investigate further the role of Cyclophilin B-PfRhopH3 interaction in the invasion of the RBC by *P. falciparum*, we next screened a dicodon polypeptide synthetic library^22, 33, 34^ to identify potential de novo peptide/proteins binders to Cyclophilin B. A bacterial two-hybrid screening was performed between the Cyclophilin B gene cloned in pTRGqq plasmid, referred as CYPBpTRGqq, and a de novo dicodon polypeptide synthetic library cloned in pBTnn plasmid referred as DIEL-pBTnn (Supplementary Fig 7g). CFP10pTRGnn/empty pBTnn and CFP10pTRGnn/ESAT6pBTnn served as the negative and positive controls, respectively^22^ (Fig. 5a, b). A 98 aa long de novo protein named CDP3 (Supplementary Table 5, Supplementary Fig. 7a) was identified as an interactor of Cyclophilin B after several rounds of segregation, re-cloning and re-transformations. We have earlier used a similar approach to identify the role of host ICAMs in cell invasion by *M. tuberculosis* and *P. falciparum* ^22^. To further explore the interaction between CDP3 and Cyclophilin B, we cloned and expressed CDP3 in pMTSA plasmid. C-terminal Histidine-tagged CDP3 was purified from *E. coli* BL21 (DE3) strain under denaturing conditions on Ni-NTA^+^ column and the protein was subsequently refolded by dialyzing it against a decreasing concentration of the denaturant. Purified and folded CDP3 protein was analyzed on SDS-PAGE and by western blot analysis (Supplementary Fig. 7b, c). Reciprocal ELISA-based interaction assays were performed between recombinant CDP3 and Cyclophilin B proteins. The binding of CDP3 with Cyclophilin B was found to be concentration dependent (Supplementary Fig. 7d). A set of wells coated with an unrelated non-specific protein HBHA did not show binding to Cyclophilin B. CDP3–Cyclophilin B interaction was further studied and quantified by SPR. CDP3 displayed a very strong interaction with Cyclophilin B, with an equilibrium dissociation constant *K*_D_ value of 7.2 × 10^−8^ M (Supplementary Fig. 7e). Since CDP3 is a binder of CypB, its binding and co-localization with CypB was evaluated on the RBC surface. Both proteins were co-localized on the RBC surface with a Pearson’s coefficient of 0.46 (Supplementary Fig 3a). We next evaluated the effect of CDP3 on the binding of Cyclophilin B with PfRhopH3-C using a bacterial three-hybrid system developed earlier by us^35^. This system allowed us to transform the “blue” two-hybrid reporter strain carrying PfRhopH3-C-pBTqq and CYPB-pTRGqq vectors with a third compatible plasmid pMTSA expressing the CDP3 protein. As the gene cloned in pMTSA vector was under the tight regulation of the arabinose-inducible pBAD promoter, triple-transformed clones were plated on X-gal plates with and without L-arabinose. As shown in Fig. 5c, d, expression of CDP3 in the presence of l-arabinose turned the PfRhopH3-C/Cyclophilin B strain from blue to white, indicating the disruption of interaction between PfRhopH3 and Cyclophilin B. Arabinose gradient liquid β-galactosidase assay further confirmed the disruption of interaction by CDP3 (Fig. 5e). The disruption was specific and quantitative as it increased with the increasing expression of CDP3 induced as a function of arabinose concentration (Fig. 5e, f). The disruption of interaction between PfRhopH3 and Cyclophilin B by CDP3 was further studied using an ELISA-based interaction assay. Incubation of Cyclophilin B coated wells with different concentrations of recombinant CDP3 protein resulted in a dose dependent reduction in PfRhopH3 binding to Cyclophilin B (Supplementary Fig. 7f). Further, we tested the efficiency of CDP3 to block the interaction between CypB and Basigin in an ELISA-based binding assay. CDP3 was unable to block the interaction between CypB and Basigin (Supplementary Fig. 8c) indicating that it is specific to the PfRhopH3-C and CypB interaction. Together, these results showed that CDP3 is an effective binder of human Cyclophilin B and further, it disrupts the hitherto strong interaction between Cyclophilin B and PfRhopH3. This potent ability of CDP3 prompted us to evaluate further the role of Cyclophilin B in *P. falciparum* invasion of host cells. As CDP3, a product of a de novo codon-shuffled library, has no homology to any known protein, we studied its secondary structural elements by circular dichroism (CD) spectroscopy. The CD spectra of the protein was analyzed using circular dichroism neural network (CDNN) tool and found to be composed of ~29% Helix and ~19% β-sheets. (Supplementary Fig. 9).

Cyclophilin B was identified as an interacting partner of PfRhopH3-C by screening the human cDNA library. However, as CypB and CypA share a high sequence similarity^15^, ELISA-based preliminary interaction studies were performed between CypA/PfRhopH3-C, CypA/CDP3, and CypA/BSG proteins. The *M. tuberculosis* ESAT6 protein was used as the negative control. Results showed that, because of high primary sequence similarity between CypA and CypB, our proteins of interest also bind to CypA (Supplementary Fig. 12).

### CypB is a crucial receptor for merozoite invasion of the RBC

**Fig. 6:**
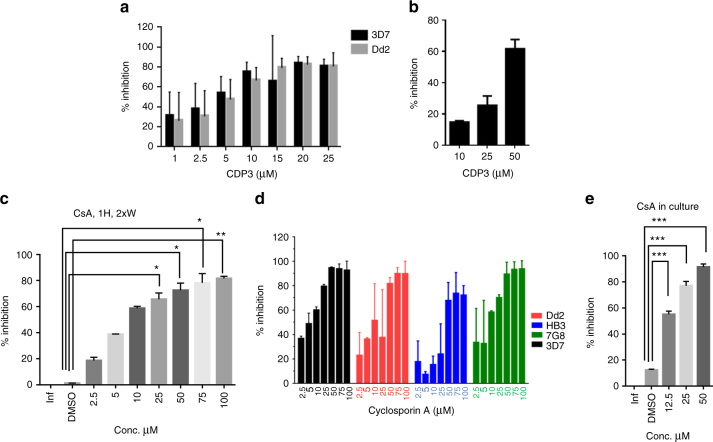
Treatment of human RBCs with CDP3 and Cyclosporin A (CsA) suggests Cyclophilin B plays a crucial role in *P. falciparum* invasion of host RBCs. Invasion Assay using CDP3: **a** Invasion inhibition of *P. falciparum* strains 3D7 and Dd2 by CDP3 into culture. Purified recombinant CDP3 protein (1–25 µM) was added to mature schizont stage parasite culture and the parasitaemia estimated after 40 h using flow cytometry. The data represent an average of three independent experiments each performed in duplicate. Invasion observed in control culture was taken as 100%. The CDP3 buffer components did not affect parasite invasion. **b** Similarly, Uninfected RBCs were treated with CDP3 (10, 25, 50 µM) for 4 h at RT followed by 3D7 merozoites infection. Parasitemia was analyzed using FACS. **c**−**e** Dose dependent inhibition of invasion by CsA treatment of RBC: Uninfected BCs treated with CsA (2.5–100 µM) followed by 3D7 merozoites infection. Invasion was reduced by ~80%. Similar results were obtained by using different strains of *P. falciparum* including Dd2, HB3 and 7G8. CsA (12.5–50 µM) added in culture at late schizont stage; parasitemia was estimated after 40 h. 80–90% inhibition of invasion was observed. Statistical significance was determined using the unpaired *t*-test with Welch’s correction. **P < 0.01 was considered significant. Each bar represents the mean ± S.D. for three independent biological replicates

As CDP3 was able to potently block the interaction between Cyclophilin B (on the human erythrocyte surface) and PfRhopH3 (on the merozoite surface), we assessed its potential to block the merozoite invasion of host RBCs using an in vitro *P. falciparum* culture. To functionally characterize the role of CDP3, different doses of CDP3 were added at 2% hematocrit to synchronized and purified *P. falciparum* (3D7 or Dd2 strains) schizont stage parasites in complete RPMI medium. After 42–48 h of incubation at 37 °C, newly formed parasites were scored by microscopic examination as well as by FACS analysis. A concentration-dependent reduction in erythrocyte invasion was observed after treatment of human RBCs with CDP3, with a maximum reduction of ~80% observed in RBCs treated with 25 μM of CDP3 (Fig. 6a). Further, an experiment was performed by treating RBCs with different concentrations of CDP3 (10, 25, and 50 µM) for 4 h. Treated RBCs were used for fresh infection with parasite and added at 2% hematocrit to synchronized and purified *P. falciparum* cultures at schizont stage in complete RPMI medium. After 42–48 h of incubation at 37 °C, newly formed parasites were scored. Concentration dependent reduction in parasitemia was observed; at 50 µM, it was found to be ~60 % (Fig. 6b).

CsA is a cyclic hydrophobic undecapeptide that binds Cyclophilins and has been shown to possess significant anti-parasitic properties including anti-malarial activity both in vitro as well as in vivo^36–38^. Although the precise anti-malarial mode of action of CsA is not yet known, CsA has been proposed to bind *Plasmodium*-encoded Cyclophilins and Cyclophilin-like proteins^39^. To confirm the role of Cyclophilins in the merozoite invasion of RBCs, and to know whether CsA acts by blocking the interaction between CypB and PfRhopH3-C, we tested its inhibitory potential in an ELISA-based inhibition assay. Importantly, CsA blocked the interaction between CypB and PfRhopH3-C (Supplementary Fig. 8a). To test whether CsA can also block the interaction between RhopH3-C and CypB in vivo, a liquid β-galactosidase assay was performed using CsA as an inhibitor. The results showed that CsA is a potent inhibitor of CypB/PfRhopH3 interaction in an in vivo system, too (Supplementary Fig. 8b). Further, we repeated the invasion assay using human RBCs treated with the drug Cyclosporin A. To find out whether Cyclophilins expressed on the human erythrocyte are important for the *P. falciparum* merozoite invasion of RBCs, human RBCs were treated with different concentrations of CsA for 1 h and treated RBCs were added at 2% hematocrit to synchronized and purified *P. falciparum* cultures at schizont stage in complete RPMI medium. After 42–48 h of incubation at 37 °C, newly formed parasites were scored. A concentration dependent reduction in the erythrocyte invasion was observed after treatment of human RBCs with CsA, with a maximum reduction of ~80% observed in RBCs treated with 100 μM of CsA (Fig. 6c). A similar dose-dependent decrease in parasitemia was witnessed with three other strains of *P. falciparum*: Dd2 (sialic acid dependent strain), HB3 (sialic acid-independent strain) and 7G8 (chloroquine resistant strain) (Fig. 6d). Simultaneously, we also tested the ability of CsA to block merozoite invasion by adding it directly in the culture during the invasion where it can bind to the RBC Cyclophilin B as well as merozoite Cyclophilins. As shown in Fig. 6e, CsA when added directly into the culture, showed better inhibitory potential; maximum reduction of parasitemia (~80%) was observed with 50 μM of CsA treatment. This indicates that upon direct addition to the culture, CsA may have bound to both, the RBC Cyclophilins as well as the merozoite Cyclophilins, in keeping with the findings of a previous report that showed reduced invasion efficiency for CsA treated merozoites^40^. These results were reproducible over three independent experiments, each carried out in duplicates.

Further, we performed an in vivo experiment to study and confirm the effect of CsA on parasitemia and survival of Balb/C mice. CsA was injected at 10 mg kg^−1^ body weight per day for four consecutive days and the dose initiated when mice were injected with 10^5^ RBCs infected with *P. berghei*. As shown in Supplementary Fig. 11a, b, CsA was able to abolish the parasitemia even till 15 days of infection as compared to the control; survival was found to be 100% 30 days post-infection. To be sure, CsA has previously been shown to cause eryptosis^41^ in the course of treatment in mice. That said, a molecular analogue similar to CsA that can specifically target the interaction between PfRhopH3 and Cyclophilin B would, we believe, be efficacious in blocking parasite invasion. Together, these results suggest Cyclophilins as clear potential targets for blocking the merozoite invasion of RBCs and advocate the development of new anti-parasitic agents based on the CsA and CDP3 scaffold.

## Discussion

In summary, we have applied a bacterial two-hybrid screening approach to identify Cyclophilin B as a novel receptor for a well-known *P. falciparum* surface protein, PfRhopH3. We confirmed the interaction between host Cyclophilin B and merozoite PfRhopH3 protein by employing a range of protein−protein interaction tools, such as protein−protein interaction assays, SPR, far-western co-immunoprecipitation and co-localization. We developed a Cyclophilin B merozoite binding assay and showed a reduction in Cyclophilin B binding on the merozoite surface in the presence of anti-PfRhopH3 antibodies, thereby confirming a receptor-ligand interaction between these two proteins. To investigate whether host Cyclophilin B does indeed play a role in merozoite invasion of human RBCs, we screened Cyclophilin B against a synthetic polypeptide library and identified a 98 amino acid long protein CDP3 that binds to Cyclophilin B and inhibits its biologically significant interaction with PfRhopH3. Importantly, addition of CDP3 inhibited the invasion of RBC by *P. falciparum* merozoites. Next, we tested the ability of the drug CsA to block the interaction between CypB and PfRhopH3 in vitro and in vivo; in both the assays CsA blocked these interactions. Further, the ability of CsA to block the host Cyclophilin B on the RBC surface was tested. We found that it efficiently blocked the merozoite invasion of treated RBCs, thereby confirming that Cyclophilin B is an important receptor for the merozoite invasion of RBCs. On the other hand, Basigin or CD147, an important integral plasma membrane glycoprotein that has been implicated in a variety of physiological and pathological activities and in particular for its role in inflammation and in merozoite invasion of human RBCs, performs these multiple functions by interacting with several human proteins or partners^1^. Basigin also functions as a signaling receptor for extracellular Cyclophilins A and B to mediate their chemotactic activities^42^. In neuron cells, interaction of CypA or CypB with Basigin has been shown to trigger a Ca^2+^ flux that results in the activation of ERK1/2 kinases^43^. Encouragingly, molecules that disrupt the interaction between Cyclophilins and Basigin are being developed for the treatment of diseases such as cancer, HIV, hepatitis C virus, and coronaviruses^44, 45^. Since Basigin has been shown to be an important receptor for merozoite invasion of RBCs^8^, we tested the co-expression and interaction of Basigin and Cyclophilin B. Both Basigin and Cyclophilin B were found to be expressed at the same time on human RBC surface and interact with each other. We further showed an interaction between their ligands PfRh5 and PfRhopH3 on merozoite surface, thereby suggesting a role of Cyclophilin B/Basigin signaling in merozoite invasion of RBCs. It is conceivable the interaction of CypB with Basigin is triggering a Ca^2+^ flux, known to be essential for *P. falciparum* merozoite invasion^46^. The parasite has been known to use multiple redundant pathways to gain entry into the RBCs^1^. We had earlier shown how dose-dependent inhibition of the RBC surface protein ICAM-4 results in blocking of the invasion process^22^. Inhibition of Basigin through anti-Basigin antibodies, as stated earlier, also leads to blocking of the parasite invasion^8^. In this context, the elucidation of a multi-protein interaction at the host-pathogen junction consisting of Cyclophilin B, Basigin, PfRhopH3, and PfRh5, the first two host proteins while the next two parasite proteins, offers an important handle for targeting parasite invasion at multiple levels. Finally, the finding that inhibitors of Cyclophilin B and Basigin are able to prevent the invasion of tested *P. falciparum* strains could provide an alternative option for future development of therapeutics.

## Methods

### Reagents

The source of bacteria and *P. falciparum* strains, as well as plasmids and antibodies is provided in Supplementary Table 2.

### Ethics statement

Group of female BALB/c mice at 4 weeks of age and 2 rabbits, obtained from ICGEB animal house facility were used for this study by following the institutional ethical committee guidelines. All animal experiments were conducted in accordance with the guidelines approved by the Institutional Animals Ethics Committee of ICGEB, New Delhi, India.

Human blood was used for *P. falciparum* culture in this study. Donor blood was obtained from Rotary blood bank (RBB), New Delhi, India. RBB is an ISO 9001:2008 certified blood bank established in 2002. It follows stringent screening procedures, careful documentation and Good Laboratory Practices for collecting, processing and testing blood.

### Dicodon library construction

The Dicodon Library^33, 35^ was prepared as described previously^22^. Briefly, a 20 µl ligation mix was prepared containing 100 ng of each of the fourteen P-5′ DNA dicodons (DCs). To this mixture was added 7.5% polyethylene glycol (v/v) and the contents heated gently to 55 °C. Once the temperature was brought down slowly to 4 °C, the DC mixture was incubated at 4 °C for a further 24 h. 100 picomol of P-5′, PAGE-purified double-stranded (ds) hairpins were added to this mixture that had been self-annealed earlier. The ligation temperature was increased to 16 °C and incubated for another 12 h. DNA precipitation and extraction from the ligation mixture was carried out using the phenol/chloroform procedure, the DNA resuspended in milli-Q water and digested with *Xba*I enzyme for 4 h at 37 °C, after which 1 μl of the digested DNA was used as a template for PCR using HP2P primer that served both as a forward and reverse primer^34^. Elution of the PCR product was carried out using DEAE membrane and the assembled DNA fragments fractionated based on their lengths (100–500 bp). The purified fragments were used as inserts for creation of de novo libraries in pBTnn and pTRGnn vectors once they had been cut with *Sna*BI and dephosphorylated.

### Human lung cDNA library

The human lung cDNA library, cloned into the pTRG vector, was acquired commercially from Stratagene, CA, USA (Catlog: 982201).

### Identification of human cyclophilin B as a binder of PfRhopH3-C

To identify host interacting partners of target *P. falciparum* RhopH3-C, a Bacterial two-hybrid experiment was performed according to the protocol provided by the manufacturer (Stratagene). Briefly, plasmid pBTnn containing the PfRhopH3-C gene and pTRG containing the Human lung cDNA library were co-transformed in R1 reporter cells. The co-transformants were plated on X-Gal indicator plates containing kanamycin (50 µg mL^−1^), chloramphenicol (30 µg mL^−1^), tetracycline (12.5 µg mL^−1^), X-Gal (80 µg mL^−1^), Isopropyl β-D-1-thiogalactopyranoside (25 µM), and phenylethyl-β-D-thiogalactoside (200 µM). A positive control was set-up in the form of ESAT6pBTnn and CFP10pTRGnn co-transformation, a well-documented *M. tuberculosis* protein-protein interaction^22, 23, 47^. Competent cells co-transformed with CFP10pTRGnn DNA and empty pBTnn plasmid represented the negative control. The indicator plates were incubated at 30 °C for 36 h. The test plates were screened for positive interactions based on development of blue colonies. The plasmids harbored in the blue colonies were segregated, confirmed using PCR, re-cloned back into their respective plasmids and co-transformed in R1 competent cells to verify the interaction. Finally, the partner pBTnn and pTRGnn plasmids were sequenced and a BLAST analysis performed to identify the interacting protein. This was found to be Human Cyclophilin B (CYPB; Peptidylprolyl Isomerase B or PPIB). Similarly, a Bacterial two-hybrid experiment was performed to identify inhibitors of chosen target proteins from de novo Dicodon libraries. Cyclophilin B (CypB) gene, cloned in plasmid pTRGqq, was used as a bait. A de novo DIEL dicodon library was cloned in pBTqq vector and the plasmids used to co-transform the R1 strain. Blue colonies obtained were analyzed in the same manner as described above and the interacting partner of CypB was identified.

### Liquid β-galactosidase assays

To confirm and quantify the intensity of protein−protein interaction, a Liquid β-Galactosidase Assay was performed as described previously^22^. Statistical significance was ascertained by performing a Student’s *t*-test. *P*-values <0.01 were considered significant.

### Bacterial three-hybrid screens

The three-hybrid screens were performed as described previously^22, 35^. Briefly, PfRhopH3-pBTqq and CYPBpTRGqq plasmids were used to co-transform the R1 reporter strain and the strain plated on X-gal indicator plates. Competent cells were prepared of the blue colony that contained both the plasmids. To set-up a bacterial three-hybrid screen, the CDP3 gene (sequence provided in Supplementary Table 6) was cloned into the pMTSA plasmid and CDP3pMTSA plasmid used to transform the R1 competent cells that contained both RhopH3-CpBTqq and CYPBpTRGqq plasmids. The transformation mixture was plated on X-gal indicator plates, both in the presence and absence of L-arabinose (1% w/v). The competent cells carrying the two plasmids, RhopH3-CpBTqq and CYPBpTRGqq, transformed with CDP3pMTSA plasmid as described above were treated as the Test set-up (that yielded white colonies in presence of l-arabinose). The same “interacting” strain when transformed with empty pMTSA plasmid served as the negative control. Plates were incubated at 30 °C. Reversion of colony color from blue to white upon induction of CDP3 expression by l-arabinose indicated disruption of RhopH3-C and CYPB interaction. The basis and validation of the bacterial three-hybrid system has been described previously^35^.

### Arabinose gradient liquid b-galactosidase assay

The three-hybrid liquid β-galactosidase assay, with increasing concentrations of l-arabinose was performed in triplicates as described previously^22, 35^. Briefly, the selected triple cotransformant and the corresponding negative control were grown to mid-log phase in the presence of 40 µM IPTG. Optical density (A600) of each culture was measured at 600 nm. Induction was carried out using varying Arabinose concentrations ranging from 0−2 mM. Growth of induced culture at 37 °C/200 r.p.m. was allowed for 3 h. Volume of 500 µl aliquot of each culture was pelleted down. Colorimetric liquid β-galactosidase assay of all samples using ONPG substrate was carried out as described previously^35^. To measure statistical significance, a Student’s *t*-test was performed. *P*-values <0.01 were considered significant. Western blot analysis of R1 cell culture lysate was performed for the expression of CDP3-His using anti-His antibody (anti-His mAb, HRP conjugated, 1 mg mL^−1^, 1: 3000 dilution).

### Cloning and purification of PfRhopH3-C

The C-terminal portion of PfRhopH3 gene (PfRhopH3-C, coding for 617–865 aa) was codon-optimized (sequence provided in Supplementary Table 6) for bacterial expression and synthesized commercially (Gene Script USA) in pUC57 vector, with the gene cloned at the unique *Sna*BI restriction site. PfRhopH3-C gene was excised and cloned in, *Sna*BI-cut dephosphorylated pMTSA plasmid. RhopH3C-pMTSA, upon induction, expresses PfRhopH3-C with a C-terminal hexa-Histidine tag (PfRhopH3-C-His6x). To obtain his-tagged PfRhopH3-C protein, *E. coli* BL21(DE3) cells harboring RhopH3-CpMTSA plasmid were grown till mid-log phase and induced with 0.5% l-arabinose for 10 h with constant shaking at 25 °C. Cells were subsequently collected and the pellet washed with PBS (pH 7.4). Cell pellet was resuspended in lysis buffer (150 mM NaCl, 2.7 mM KCl, 4.3 mM Na_2_HPO_4_, 1.47 mM KH_2_PO_4,_ pH 8.0, 2 mM PMSF) and sonicated till a clear lysate was obtained. The lysate was centrifuged at 17,000×*g* for 1 h and the obtained pellet solubilized in solubilization buffer (150 mM NaCl, 2.7 mM KCl, 4.3 mM Na_2_HPO_4_, 1.47 mM KH_2_PO_4_, 8 M urea, pH 8.0) and incubated at 25 °C for 12 h with continuous shaking. Solubilised sample was centrifuged at 20,000×*g* for 1 h at 25 °C. The resultant supernatant filtered through 0.45 μm nitrocellulose filter and allowed to bind overnight to 10 mL of Ni-NTA superflow resin (50% (v/v) suspension in 25% alcohol) at 25 °C with shaking at 150 r.p.m. The column was washed with 10-bed volumes of binding buffer (150 mM NaCl, 2.7 mM KCl, 4.3 mM Na_2_HPO_4_, 1.47 mM KH_2_PO_4_, 8 M Urea, 10 mM imidazole, pH 8.0) and the bound protein eluted from Ni-NTA by setting up an imidazole-based gradient between buffer A (150 mM NaCl, 2.7 mM KCl, 4.3 mM Na_2_HPO_4_, 1.47 mM KH_2_PO_4_,8 M urea, 10 mM imidazole, pH 8.0) and buffer B (150 mM NaCl, 2.7 mM KCl, 4.3 mM Na_2_HPO_4_, 1.47 mM KH_2_PO_4_,8 M urea, 500 mM imidazole, pH 8.0). The purification was performed using an AKTA purifier, GE, USA. The pooled eluate was dialyzed against 150 mM NaCl, 2.7 mM KCl, 4.3 mM Na_2_HPO_4_, 1.47 mM KH_2_PO_4_, pH 8.0, by gradual removal of urea from 8−0 M. Purified protein was analyzed by running on SDS-PAGE and western blot using anti-PfRhopH3 antibody (Rabbit polyclonal (1:5000), Supplementary Fig. 2a)). Purified PfRhopH3-C protein used to raise antibody as described in Supplementary methods.

### Cloning and purification of CDP3 protein

CDP3 gene was PCR-amplified using the HP2PS1 primer (Supplementary Table 3), digested with *Sna*BI restriction enzyme, and cloned into *Sna*BI-digested pMTSA vector to yield plasmid CDP3pMTSA. CDP3pMTSA was used to transform *E. coli* BL21(DE3) and the culture grown under 0.2% l-arabinose induction for 4 h with constant shaking at 37 °C in the presence of Streptomycin (50 mg mL^−1^). CDP3 was expressed as a fusion protein with 6X-His tag at its C-terminal end. Cells were collected and the pellet washed with 1XPBS (pH 7.4), following which it was resuspended in lysis buffer (50 mM HEPES 150 mM NaCl, pH 7.4, 2 mM PMSF) and sonicated till a clear lysate was obtained. The lysate was centrifuged at 17,000×*g* for 1 h and the obtained pellet solubilised in solubilization buffer (50 mM HEPES, 150 mM NaCl, 8 M urea, 5 mM imidazole, pH 7.4) and kept overnight at room temperature with continuous shaking. Solubilised sample centrifuged at 20,000×*g* for 1 h at 25 °C. The resultant supernatant was filtered through 0.45 μm nitrocellulose filter and allowed to bind overnight to 10 mL of Ni-NTA superflow resin (50% (v/v) suspension in 25% alcohol) at 25 °C with shaking at 150 r.p.m. The column was washed with 10-bed volumes of binding buffer (50 mM HEPES, 150 mM NaCl, 8 M urea, 5 mM imidazole, pH 7.4) and the bound protein eluted by setting up an Imidazole-based gradient between buffer A (50 mM HEPES, 150 mM NaCl, 8 M urea, 5 mM imidazole, pH 7.4) and buffer B (50 mM HEPES, 150 mM NaCl, 8 M urea, 500 mM imidazole, pH 7.4). The purification was performed using an AKTA purifier. The pooled eluate was dialyzed against 10 mM HEPES, 150 mM NaCl, pH 7.4 by gradual removal of urea from 8 to 0 M. Purified protein was analyzed on SDS-PAGE and western blot using anti-His antibody ((anti-His mAb, HRP conjugated, 1:3000 dilution), Supplementary Fig. 7b). Purified CDP3 was analyzed for the presence of Endotoxins using LAL (Limulus amebocyte lysate) gel clot assay kit provided by Charle’s River and it was found to be free of endotoxins.

### In vitro protein−protein interaction studies

Recombinant proteins Cyclophilin B (Catlog: 11004-H08H-100) and Basigin (BSG/CD147, Catlog: 10186-H08H-100) were obtained commercially from Sino Biological Inc., China (Supplementary Fig. 10a, b). To check the specificity of antibodies that have been used in present study, western blot analysis was performed to detect the native proteins (Supplementary Figs. 2c, 10c–g, Supplementary Table 2).

### Far-western

Far-western blotting experiments were performed according to the protocol described previously^48^ with minor modifications. Briefly, bait proteins PfRhopH3-C and Cyclophilin B (2 µg each) were run on 12% SDS-PAGE and transferred on to PVDF membrane. Denaturation and renaturation of bait proteins on membrane was carried out according to the protocol. Prey proteins CypB and BSG (3 µg mL^−1^ each) in binding buffer (100 mM NaCl, 20 mM Tris (pH 7.6), 0.5 mM EDTA, 10% glycerol, 0.1% Tween-20, 2% skim milk powder, 1 mM DTT) were overlaid on renatured bait proteins PfRhopH3-C and CypB, respectively, for 2 h at room temperature (RT). After washing, detection for prey proteins was carried out using the antibodies: anti-Cyclophilin B (Rabbit polyclonal, 1:2000) and anti-Basigin (Mouse monoclonal, 1:2000), respectively, by incubating overnight at 4 °C. Secondary antibodies: goat anti-Rabbit IRDye 800CW (1:15,000) or goat anti-mouse IRDye 800CW (1:15,000) were overlaid on to the membrane for 1 h at RT and the membrane image captured using LI-COR Odyssey FC Instrument. *M. tuberculosis* proteins, HBHA (Heparin binding Heme-agglutinin; prepared in-house; unpublished work) and CFP-10^23^ were used as negative control.

### Co-immunoprecipitation

Co-Immunoprecipitation was performed according to the protocol provided by Pierce Co-Immunoprecipitation (Co-IP) Kit, Thermo Fisher Scientific, with slight changes. Briefly, antibodies against bait proteins PfRh5 and CypB were immobilized on the coupling resin using immobilization buffer at room temperature for 1 h, following which the mixture of bait and prey (PfRhopH3-C) proteins, 8 µg each were allowed to bind on to immobilized antibodies. This binding was allowed for up to 4 h on 4 °C. Subsequent to stringent washing of the reaction mixture, elution of bound protein was carried out using 30 µl elution buffer, and 15 µl the eluted protein mixture analyzed by SDS-PAGE followed by western blotting. Identification of prey protein PfRhopH3-C was achieved through the use of rabbit anti-PfRhopH3-C antibody (1:5000 dilution) followed by anti-rabbit HRP conjugated secondary antibody. ImmunoCruz western blotting luminol reagent (Santa Cruz Biotechnology) used as substrate for HRP and images were acquired using FluorChem M (protein simple) western blotting system. Mycobacterial CFP10 protein was used as the negative control bait protein (Supplementary Fig. 5f).

### ELISA

Elisa based analysis of protein interactions was performed as described previously^22, 49^ with slight modifications. Briefly, the bait proteins PfRhopH3-C, Cyclophilin B, Basigin, and CDP3 were coated overnight on a 96-well ELISA plate (NuncMaxisorb ELISA plates) at 200 ng concentration, in 100 µl of 0.1 M carbonate/bicarbonate-coating buffer, pH 9.6. Coated wells were blocked using the blocking buffer (1XPBS, 0.1% Tween-20 and 3% BSA) for 1 h at 37 °C, following which the plates were washed three times with washing buffer (1XPBST) and incubated for 1 h at 37 °C with the corresponding prey proteins (interacting partners): Cyclophilin B, Basigin, PfRh5, and Cyclophilin B respectively by gradually increasing their concentration (100 ng, 200 ng, 400 ng, and 800 ng) in 100 µl of binding buffer (50 mM HEPES, 250 mM Potassium acetate and 5 mM magnesium acetate, pH 8.0). Following stringent washing (three times with the washing buffer), primary antibodies against the different prey proteins, anti-CypB (rabbit polyclonal, 1:2500), anti-CD147 (mouse monoclonal, 1:2000) and ant-PfRH5 (rabbit polyclonal, 1:10,000) were added to the respective wells and the mixture incubated at 37 °C for 1 h. For the negative control, non-specific antibodies (anti-CFP10 rabbit polyclonal and anti-ESAT6 mouse polyclonal, 1:2000 dilutions each) were used. Subsequent to washing, secondary antibodies: anti-rabbit and anti-mouse (HRP conjugated) were added (1:3000 dilutions) and the plates incubated further for 1 h. The plates were washed three times with washing buffer (1XPBST) and further three times with 1XPBS, following which was added 100 µl of HRP substrate TMB and the plates incubated at 37 °C for 30 min. Finally, stop solution (1N H_2_SO_4_) was added and the optical density measured at 450 nm using an ELISA plate reader. Mycobacterial HBHA and CFP10 proteins were used as negative controls for ELISA.

### Surface plasmon resonance

SPR assays were performed according the protocol described previously^22^ on BIACORE T200 instrument (GE Healthcare) at 25 °C, using HEPES buffer (50 mM HEPES, 150 mM NaCl and 5 mM MgCl_2_, pH 7.4) as the running buffer and acetate buffer (pH 4.0) as the immobilization buffer.

### Cyclophilin B interactions by SPR

For these experiments, CypB (20 µg mL^−1^; 200 RU (response units)) was immobilized on to an S-series CM5 sensor chip using amine coupling surface chemistry (GE Healthcare). Acetate buffer (pH 4.0) used as the immobilization buffer. For measuring kinetics of interaction, increasing concentrations of recombinant PfRhopH3-C, BSG, and CDP3 were injected at a flow rate of 30 µl/min over the immobilized CypB as well as the reference flow cell, separately. The surfaces were regenerated with a pulse of 10 mM glycine at pH 3.0 at the end of each injection cycle. Duplicate injections of the same concentration in each experiment were super-imposable, demonstrating no loss of activity after surface regeneration. Reference-subtracted sensorgrams were analyzed using the Biacore evaluation software 4.1.1 (GE Healthcare). Kinetic parameters of the interaction were determined by plotting binding responses in steady-state region of the sensorgrams, against analyte concentration, and fitted to the standard 1:1 (Langmuir) bimolecular interaction with simultaneous fitting of *k*_a_ and *k*_d_. Kinetic constants were calculated through nonlinear regression fitting to the association and dissociation phases of the sensorgrams.

### Interaction of PfRh5 and PfRhopH3-C by SPR

200 RU of PfRh5 (20 µg mL^−1^) was immobilized on to an S-series CM5 sensor chip as described above. Acetate buffer (pH 4.0) was used as the immobilization buffer. For measuring kinetics of interaction, increasing concentrations of recombinant PfRhopH3-C were injected, over immobilized PfRh5 as well as the reference flow cell, at a flow rate of 30 µl/min. Surface regeneration and kinetics analyses were performed using the methods described above. The PfClpQ^50^ protein was used as negative control for the SPR experiments (Supplementary Fig. 6).

### *Plasmodium falciparum* culture and merozoite preparation

*P. falciparum* 3D7, Dd2, HB3, and 7G8 strains (Supplementary Table 2) were cultured in complete RPMI (RPMI 1640 (Invitrogen, USA)), 50 mg L^−1^ hypoxanthine (Sigma, USA), 0.5 g L^−1^ Albumax I (Gibco, USA) and 2 g L^−1^ sodium bicarbonate (Sigma, USA) using O^+^ human erythrocytes (4% haematocrit) under mixed gas (5% O_2_, 5% CO_2_, and 90% N_2_) conditions. Cultures were synchronized in early ring stage with 5% sorbitol for at least two successive cycles. Synchronized and healthy parasites at schizont stage were purified by percoll sedimentation. *P. falciparum* merozoites were isolated from infected erythrocytes as described earlier^51, 52^. Briefly, 12–15% tightly synchronized schizont stage culture was layered on 65% percoll and centrifuged at 2000 r.p.m. for 20 min at 4 °C. The inter-phase of infected erythrocytes was recovered and washed twice in incomplete RPMI. These purified schizont stage parasites were grown again in the presence of E64 for 6–8 h, allowing schizonts to mature without rupturing the erythrocyte membrane. The membrane of erythrocytes was ruptured by passing through a 1.2 μm Acrodisc 32-mm syringe filter (Pall) and purified merozoites were recovered by centrifugation at 4000×*g* for 10 min at RT.

### Localization of CypB and basigin on the RBC surface

Uninfected O^+^ human erythrocytes were washed twice in 1XPBS and fixed in PFG (4% paraformaldehyde +0.0075% glutaraldehyde) for 20 min at room temperature. Fixed erythrocytes were washed again in 1XPBS and incubated with blocking buffer (4% BSA) for 2 h at RT on shaker. These erythrocytes were incubated with monoclonal anti-CypB (mouse; 1:50) and polyclonal anti-Basigin (rabbit; 1:50) antibodies for 2 h on ice. After washing with blocking buffer, erythrocytes were incubated with alexa-fluor 488 conjugated goat anti-mouse IgG antibody (1:200) for CypB and alexa-fluor 594 conjugated goat anti-rabbit IgG antibody (1:200) for Basigin for 1 h at room temperature. The erythrocytes were washed, smeared on slides and analyzed using a Nikon A1-R confocal microscope.

### Interaction of basigin and cyclophilin B with merozoites

Purified merozoites were incubated with appropriate (25 µM) concentrations of Cyclophilin B and Basigin proteins for 2 h at RT on a rotator. After washing with PBS, merozoites were fixed with fixation solution (PFG) for 20 min at RT followed by blocking in blocking buffer (4% BSA) for 2 h at RT. These merozoites were then mounted with monoclonal anti-CypB (mouse; 1:50) and anti-BSG (rabbit; 1:50) antibodies for 2 h on ice. Merozoites were washed with blocking buffer and incubated with alexa-fluor 488 conjugated goat anti-mouse IgG antibody (1:200) for CypB and alexa-fluor 594 conjugated goat anti-rabbit IgG antibody (1:200) for BSG for 1 h at room temperature. DAPI was added during the last 10 min of this incubation period. The merozoites were washed again, smeared on slides and analyzed using a Nikon A1-R confocal microscope. In order to study the specificity and role of PfRhopH3 in CypB interaction, merozoites were pre-incubated with rabbit anti-PfRhopH3 antibody and anti-Rap2b (1:25) separately for 15 min followed by the addition of 25 µM of Cyclophilin B. Binding of Cyclophilin B was assessed by incubating these merozoites with alexa-fluor 488 conjugated goat anti-mouse IgG antibody (1:200) for CypB. The fluorescent intensity of the CypB binding on merozoite surface was measured by using intensity profile setting on NIS element software.

### Effect of cyclosporin A on invasion

To assess the effect of Cyclosporin A (CsA) on *P. falciparum* growth, synchronous cultures at late schizont stage with parasitemia of 1% were treated with varying concentrations (12.5, 25, and 50 µM) of CsA in a 96-well culture plate in duplicate. Uninfected erythrocytes, infected erythrocytes alone, and infected erythrocytes treated with DMSO were taken as controls. Parasites were maintained for 40 h and stained with ethidium bromide (EtBr, 10 µg ml^−1^). 100,000 total events were acquired per sample, using Cell Quest software on a FACS Caliber flow cytometer. In parallel, uninfected erythrocytes were treated first with CsA at different concentrations (2.5–100 µM) for 1 h at RT on a rotator and washed twice with incomplete RPMI media. Treated erythrocytes were used for fresh infection maintaining 2% hematocrit and 1% parasitemia. Untreated erythrocytes and DMSO-treated erythrocytes were used as controls.

### Effect of CDP3 on invasion

To evaluate the potential of CDP3 protein on parasite invasion inhibition in chloroquine sensitive, 3D7, and resistant Dd2 strains, synchronized parasites at schizont stage were seeded in 96-well culture plates maintaining 1% parasitemia and 2% haematocrit. Recombinant purified endotoxin free CDP3 was added to the culture medium at varying concentrations of 1, 2.5, 5, 10, 15, 20, 25 μM and the culture plate incubated at 37 °C. After 40 h, the parasites were stained with EtBr and the parasitemia calculated using FACS. Uninfected erythrocytes and infected erythrocytes alone were taken as controls.

In another experiment, uninfected RBCs were treated with different concentrations (10, 25, and 50 μM) of CDP3 for 4 h at RT. After removal of unbound CDP3, treated erythrocytes were used for fresh infection maintaining 2% hematocrit and 1% parasitemia. Uninfected erythrocytes and infected erythrocytes alone were taken as controls.

### Erythrocyte binding assay of PfRhopH3-C

Uninfected human erythrocytes were washed with incomplete RPMI media and incubated with 20 μM each of PfRhopH3-C and MLH (*Plasmodium* nuclear helicase) protein in incomplete RPMI media followed by fixation with PFG and subsequent blocking with BSA. Cells were incubated with anti-RhopH3-C rabbit sera and then with anti-rabbit alexa-flour 594 secondary antibody and DAPI. Cells were visualized using Nikon A1-R confocal microscope and the images were processed using NIS elements.

### In Vivo study of cyclosporin A in the murine model

Cryopreserved infected RBC blood from *P. berghei* infected mice were revived by injecting it intraperitoneally into Balb/C mice. Percentage of parasitemia was determined from Giemsa-stained blood smears prepared from the tail region. After achieving 10−12% parasitemia, 4-week-old Balb/C mice were reinjected with 10^5^ iRBCs and mice were divided into two groups (five mice in each group). One group of mice was injected with buffer control and the second group of mice with CsA at 10 mg kg^−1^ body weight of mice. Doses were initiated from the day zero of infection and followed up to day 4. The blood smears of treated mice were prepared from day 4 and mice were observed till day 30 for survival.

### Data availability statement

The data supporting the findings of this study are available from the authors on request.

## Electronic supplementary material

Supplementary Information
