# Supplementary material for: Human Cyclophilin B forms part of a multi-protein complex during erythrocyte invasion by Plasmodium falciparum
Source: Nat Commun. 2017 Nov 16;8:1548. doi: 10.1038/s41467-017-01638-6 (PMC5691159; doi:10.1038/s41467-017-01638-6)
Supplement: Supplementary file 1 — Supplementary Information [file 41467_2017_1638_MOESM1_ESM.pdf]

Supplementary Information

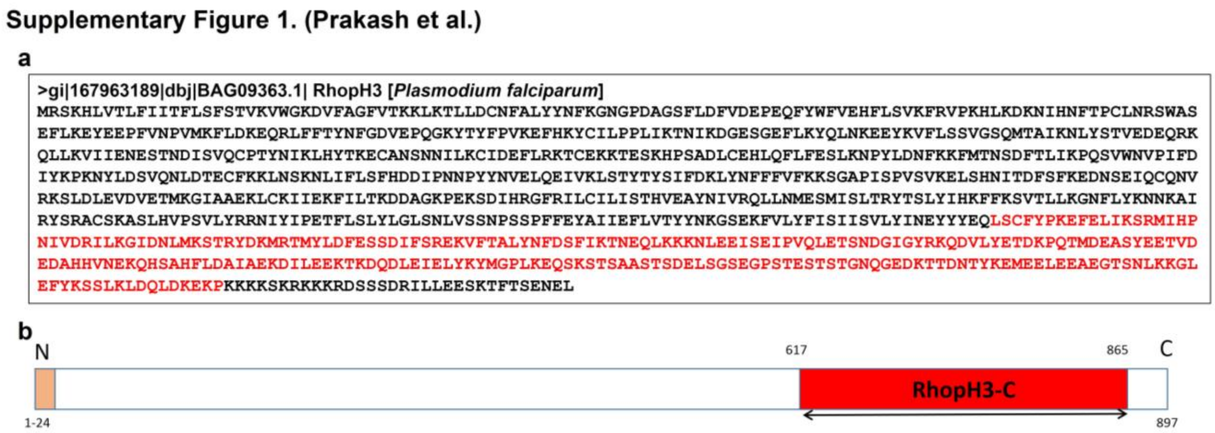

Supplementary Figure 1. Sequences of RhopH3 protein (a). Complete sequence of the 897 amino acid long PfRhopH3 protein. Sequence in red indicates the C-terminal PfRhopH3-C fragment chosen for the present study. **(b)** Schematic of the PfRhopH3 protein. 1-24 aa: signal sequence; 617-865 aa, red box: PfRhopH3-C.

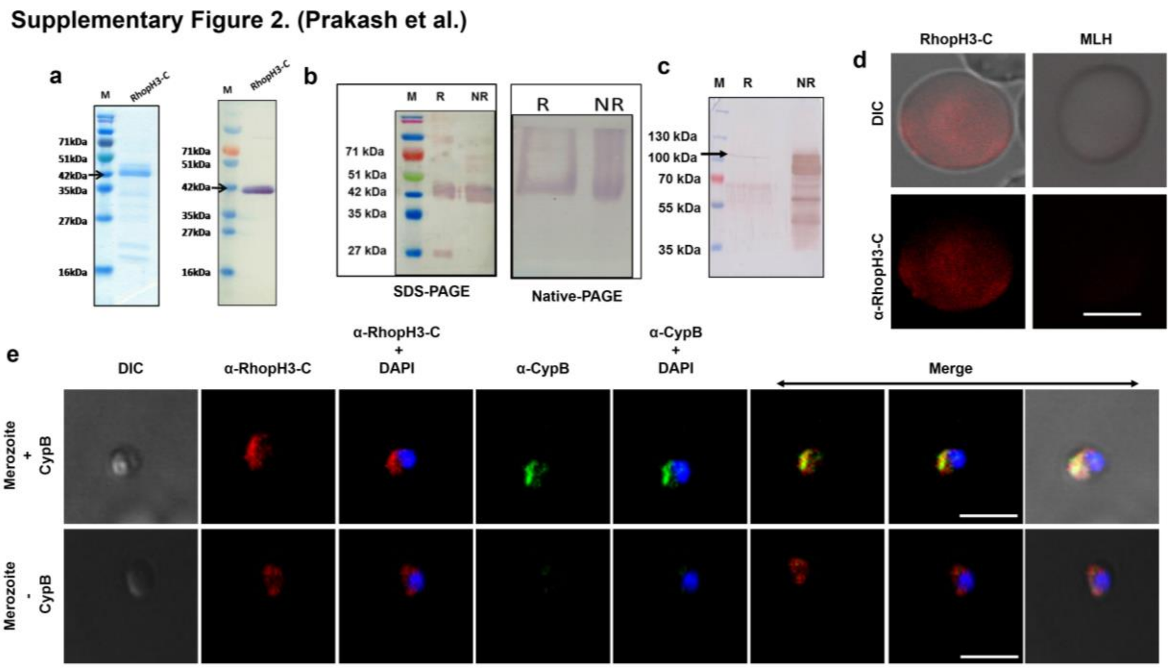

Supplementary Figure 2. Expression and purification of PfRhopH3-C protein and its binding and co-localization, on the RBC surface as well as on the merozoite surface. PfRhopH3-C gene was codon-optimized for expression in *E. coli* and cloned in expression plasmid pMTSA under the control of L-arabinose inducible pBAD promoter. *E.coli* BL21 (DE3) competent cells were transformed with the plasmid and induced with 0.5% L-arabinose at 25 ºC for 10 h. Protein was found to be expressed as inclusion bodies. Purification was achieved under denaturing conditions and the protein was dialysed to remove the denaturing agent. **(a)** Purified protein was analysed on SDS-PAGE and Western blot using anti-RhopH3-C antibody. **(b)** Purified and refolded recombinant PfRohopH3-C protein showed difference of mobility on SDS-PAGE and Native-PAGE under reduced (R) and non-reduced (NR) conditions. **(c)** Western blot analysis of native PfRhopH3 protein in schizont lysate by anti-PfRhopH3-C polyclonal antibody (rabbit) under reducing (R) and non-reducing (NR) conditions. **(d)** PfRhopH3-C binds to the RBC surface. Uninfected human erythrocytes were washed with

incomplete RPMI media and incubated with 20 μM each of PfRhopH3-C and MLH (*Plasmodium* nuclear helicase) protein in incomplete RPMI media followed by fixation with paraformaldehyde and subsequent blocking with 3% BSA. Cells were incubated with anti-PfRhopH3-C antibody (rabbit polyclonal) followed by anti-rabbit alexa-fluor 594 secondary antibody. **(e)** Binding and co-localization of CypB with PfRhopH3 on merozoite surface. **Top panel:** Merozoites were incubated with 20 μM CypB protein. Subsequently, merozoites were stained with anti-RhopH3-C rabbit and anti-CypB mouse antibody and probed with anti-rabbit alexa-fluor 594 and anti-mouse alexa-fluor 488 secondary antibodies, respectively. PfRhopH3 and CypB co-localized on the surface of human RBC with Pearson's coefficient of 0.58. **Bottom panel**: Merozoites without incubation with the CypB protein. Scale bar = 5 μm.

Supplementary Figure 3. (Prakash et al.)

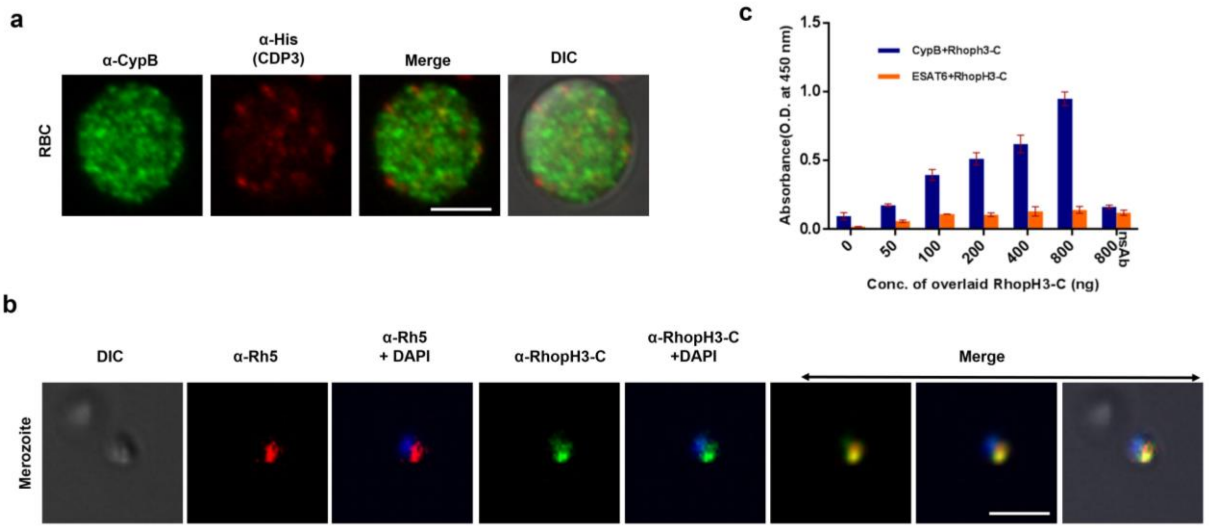

Supplementary Figure 3. Binding and co-localization of CDP3 protein with CypB on the RBC surface and co-localization of PfRhoH3 and PfRh5 on the merozoite surface. **(a)** Co-localization of CDP3-His protein with CypB expression on the RBC surface. Uninfected RBCs were treated with 20 µM of CDP3 protein and incubated with anti-His (CDP3) rabbit and anti-CypB mouse antibody. Subsequently, alexa-fluor 594 anti-rabbit and alexa-fluor 488 anti-mouse were used to detect CDP3 and CypB respectively. (**b)** Co-localization of PfRh5 and PfRhoph3-C: Merozoites were stained with anti-PfRh5 antibody (Rabbit) and anti-PfRhopH3 antibody (mouse) and subsequently probed with anti-rabbit alexa-fluor 594 and anti-rabbit alexa-fluor 488 secondary antibodies respectively. Pearson`s coefficient of col-localization is 0.62. **(c)** Reciprocal ELISA between CypB and RhopH3-C to confirm the interaction. CypB was coated on the ELISA plate and subsequently overlaid with RhopH3-C. Binding of

RhopH3-C was detected using anti-RhopH3-C antibody (rabbit). Each bar represents the mean ± S.D. for triplicate experiment. Scale bar = 5 µm.

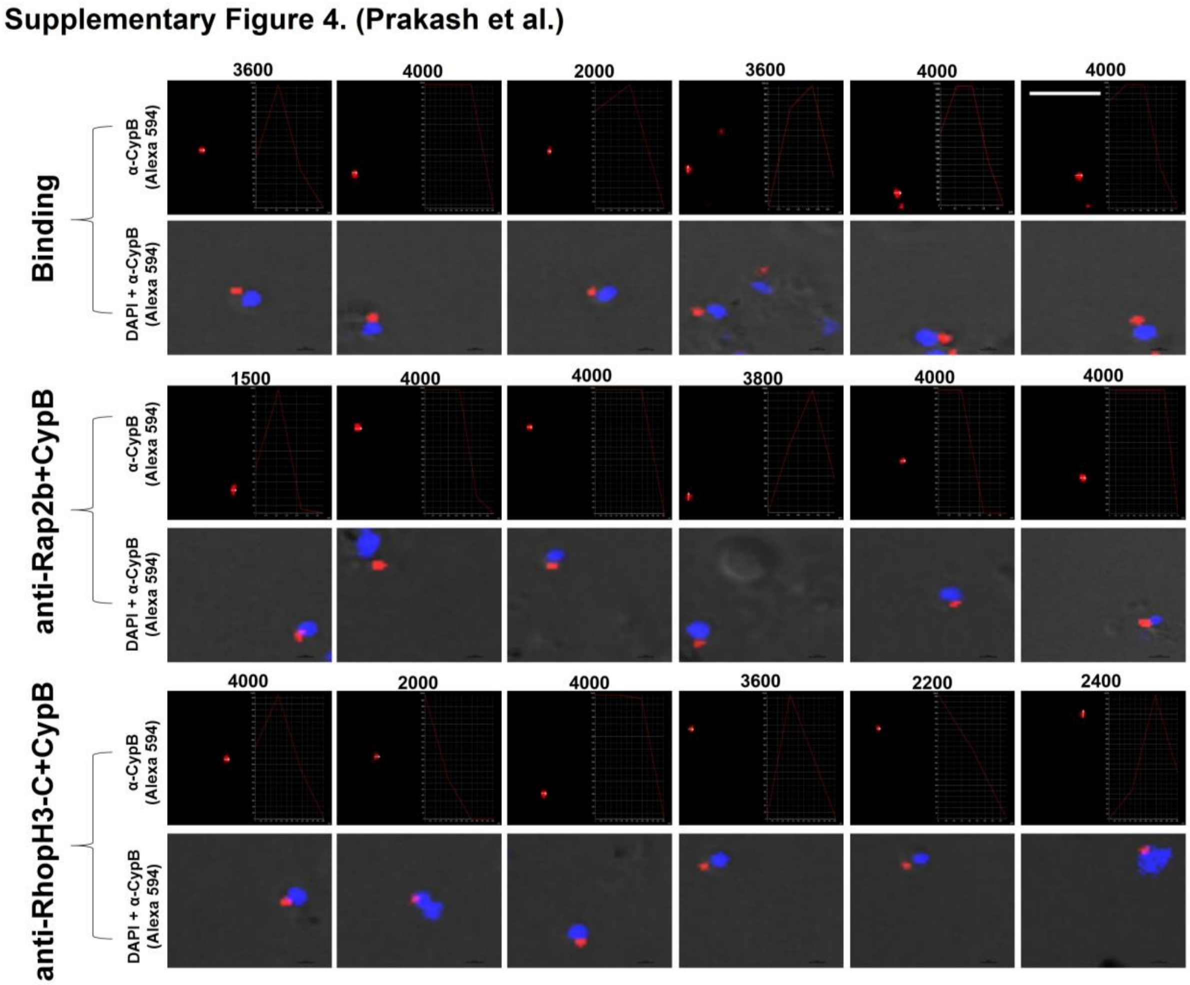

Supplementary figure 4. Binding assay of CypB on merozoite. Merozoite intensities that are highlighted with blue color in Supplementary Table 1 are presented here. Panel named **"binding"** shows the CypB binding on the Merozoite surface without any treatment with antibody; **"anti-Rap2b+CypB"** panel represents the binding of CypB on merozoite after treatment with anti-Rap2b antibody; **"anti-RhopH3-C+CypB"** panel

represents the binding of CypB protein after treatment of merozoite with anti-RhopH3-C antibody. Intensity for each merozoite is mentioned at the top. Scale bar = 5 µm.

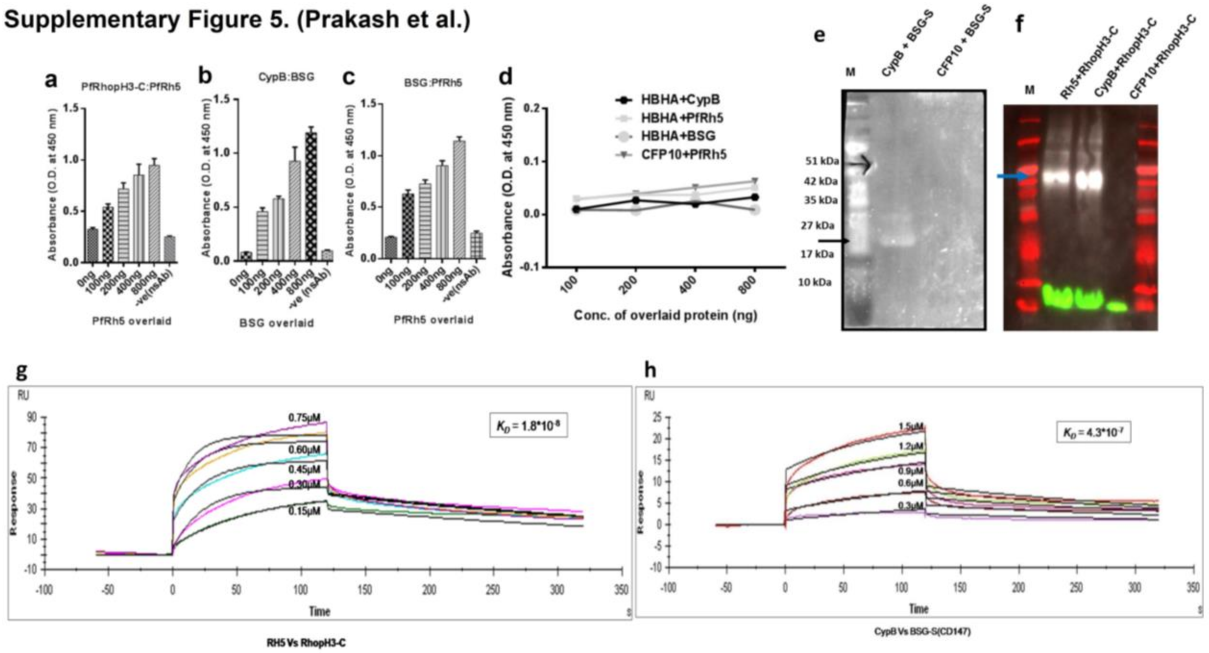

Supplementary Figure 5. ***In vitro*** **protein-protein interaction assays to confirm the interaction of proteins involved in complex formation during invasion.** ELISA-based interaction between **(a)** PfRhopH3 and PfRh5; **(b)** CypB and BSG; and **(c)** PfRh5 and BSG. 200 ng each of the bait proteins PfRhopH3-C, CypB and BSG (CD147) were coated NuncMaxisorb ELISA plates overnight at 4 ºC. Subsequently, corresponding prey proteins PfRh5, BSG, and PfRh5 were overlaid with increasing concentrations as listed and detected by anti-PfRh5 (rabbit polyclonal) and anti-BSG (rabbit polyclonal) antibodies, followed by anti-rabbit HRP conjugated secondary antibody. A nonspecific antibody, anti-CFP10 (rabbit polyclonal) was used as the antibody control. Each bar represents the mean ± S.D. for triplicate experiment. **(d)** Negative control for ELISA experiments. Nonspecific proteins: 200 ng of Mycobacterial HBHA and CF10 were

coated on ELISA plate and overlaid with CypB, Basigin, and Rh5 respectively. No interaction was observed between coated and overlaid proteins. **(e)** Far western confirming the interaction between CypB and BSG. Detection of BSG (indicated by arrow) was carried out using anti-BSG antibody (mouse monoclonal) and anti-mouse IRDye 800CW (1:15000) secondary antibody. Membrane was imaged through LI-COR Odyssey FC Instrument. Mycobacterial CFP10 was used as the negative control protein **(f)** Co-immunoprecipitation showing interaction between PfRh5/PfRhopH3-C and CypB/PfRHopH3-C. Mycobacterial CFP10 used as negative control. **(g) & (h)** SPR-based interaction analysis of PfRh5:PfRhopH3-C and CypB:BSG pairs. CypB and PfRh5 were immobilized on separate flow cells of the CM5 sensor chip and their respective analytes PfRhopH3-C and PfRh5 injected in increasing concentrations. Reference-subtracted binding data was plotted as a binding curve and the equilibrium dissociation constant calculated using non-linear regression fitting of a simple Langmuir binding isotherm to the data.

Supplementary Figure 6. (Prakash et al.)

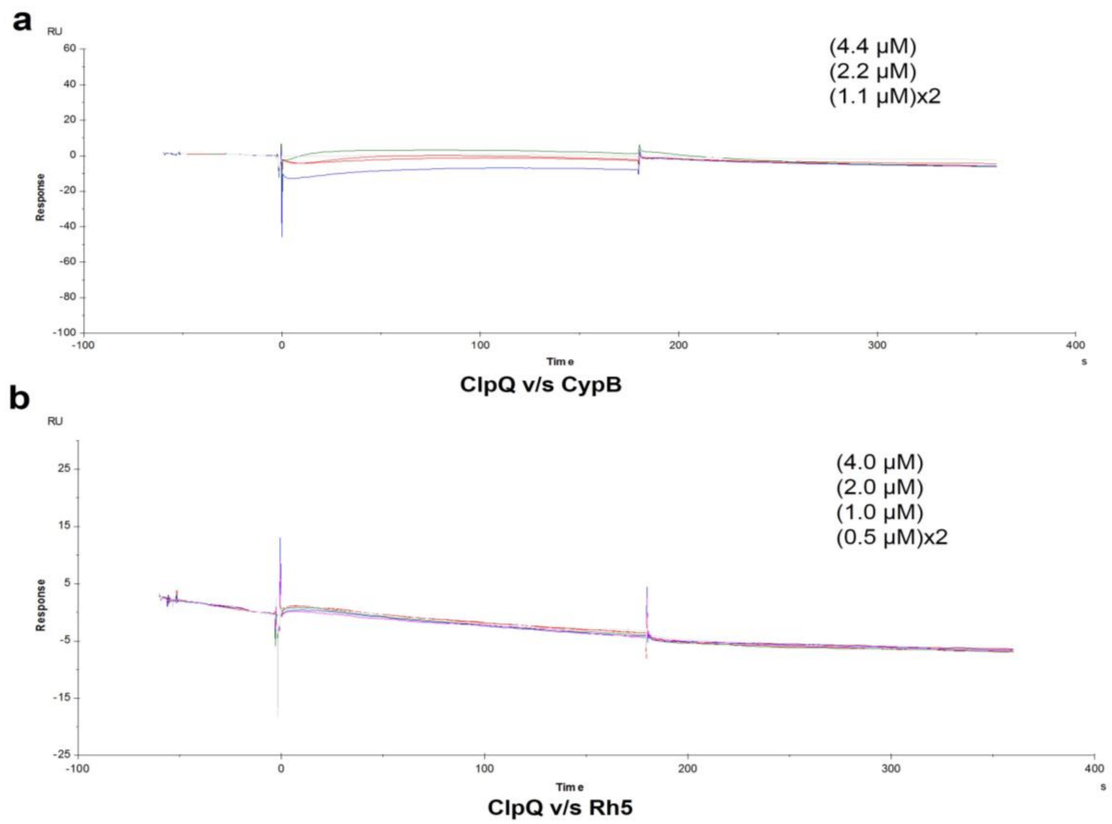

Supplementary Figure 6. Negative control for SPR experiments.

The proteins that were captured/immobilized on the S-series CM5 sensor chip were PfRh5 and CypB. The SPR analysis of these captured proteins was carried using a non-specific protein ClpQ as the ligand (Immobilized), and **(a)** CypB and **(b)** PfRh5 as the analyte(s). Analytes were run over immobilized proteins in running buffer. Both PfRh5 and CypB showed negligible response and were not found to be interacting with ClpQ.

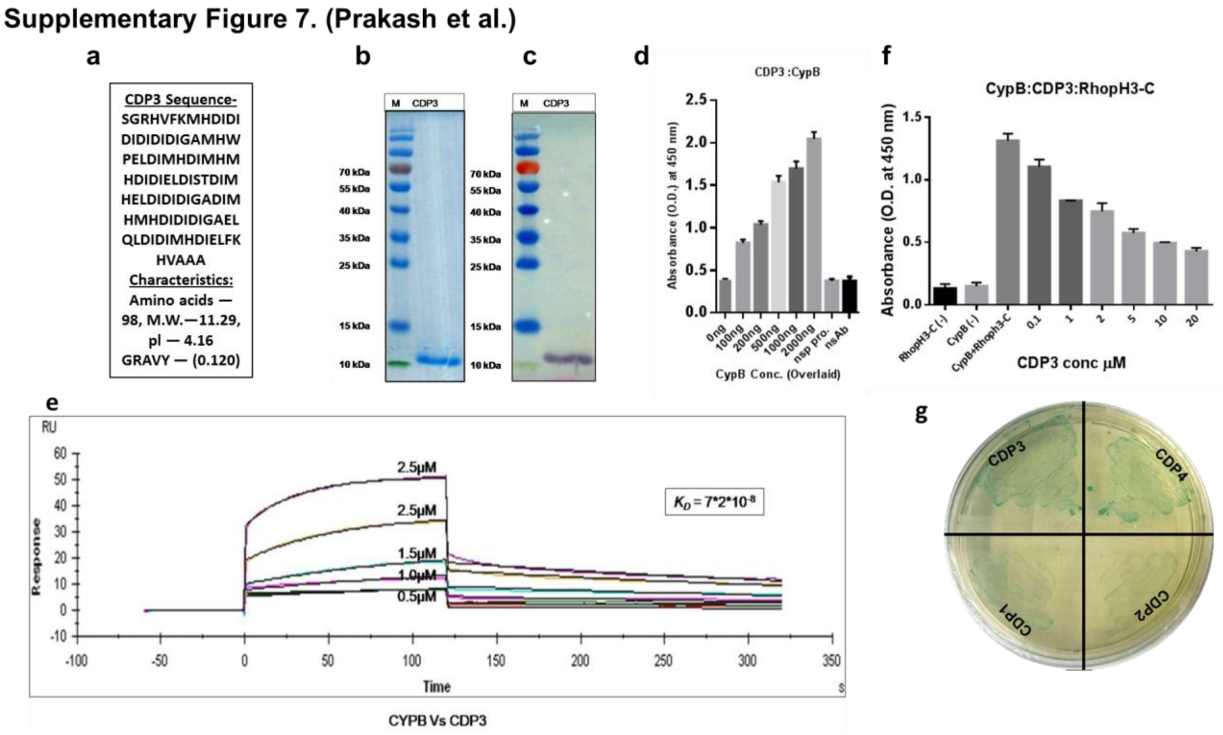

Supplementary Figure 7. Expression, Purification and *In vitro* Protein-Protein interaction assays between CypB and CDP3. (a) Amino-acid sequence of the identified *de novo* CDP3 polypeptide. Protparam tool from the ExPASy server (http://web.expasy.org/protparam/) was used to ascertain MW, pI and hydropathicity of CDP3. **(b)** CDP3 gene was cloned in expression plasmid pMTSA under the control of L-arabinose inducible pBAD promoter. *E.coli* BL21 (DE3) competent cells were transformed with the plasmid and induced with 0.2% L-arabinose at 37 ºC for 4 h. Protein was found to be expressed as inclusion bodies. Purification was achieved under denaturing conditions and the protein dialysed to remove the denaturing agent. SDS-PAGE and **(c)** Western blot analysis of purified recombinant CDP3 protein. Western blot was carried out using anti-His antibody, HRP conjugated (1:3000) **(d)** ELISA confirming the interaction between CDP3 and CypB. 200 ng of CDP3 was coated on NuncMaxisorb ELISA plate overnight at 4 ºC. CypB was overlaid with the CypB

concentrations listed. Detection was achieved using anti-CypB antibody (rabbit polyclonal), followed by HRP conjugated secondary antibody. A nonspecific protein (HBHA) and a nonspecific antibody were used as negative controls. Each bar represents the mean ± S.D. for triplicate experiment. **(e)** SPR to assess the kinetics of interaction between CypB and CDP3. CDP3 was injected over immobilized CypB in increasing concentrations. K_D was found to be 7.2×10^{-8} M **(f)** ELISA based inhibition of interaction between CypB and PfRhopH3-C using CDP3 as an inhibitor. 200 ng of CypB protein was coated on NuncMaxisorb ELISA plate overnight at 4 ºC. Next, the CDP3 protein was overlaid in increasing concentrations as listed, with an incubation time of 1 h. Finally, 200 ng of PfRhopH3-C was overlaid and incubated for 1 h at 37 ºC. PfRhopH3-C was detected using anti-PfRhopH3-C antibody (rabbit, 1:5000) followed by HRP conjugated secondary antibody. Optical density was measured at 450 nm. Each bar represents the mean ± S.D. for triplicate experiment. **(g)** Liquid patching of the blue colonies obtained during the screening of DIEL di-codon library against CypB. Intensity of blue color indicates the strength of interaction.

Supplementary Figure 8. (Prakash et al.)

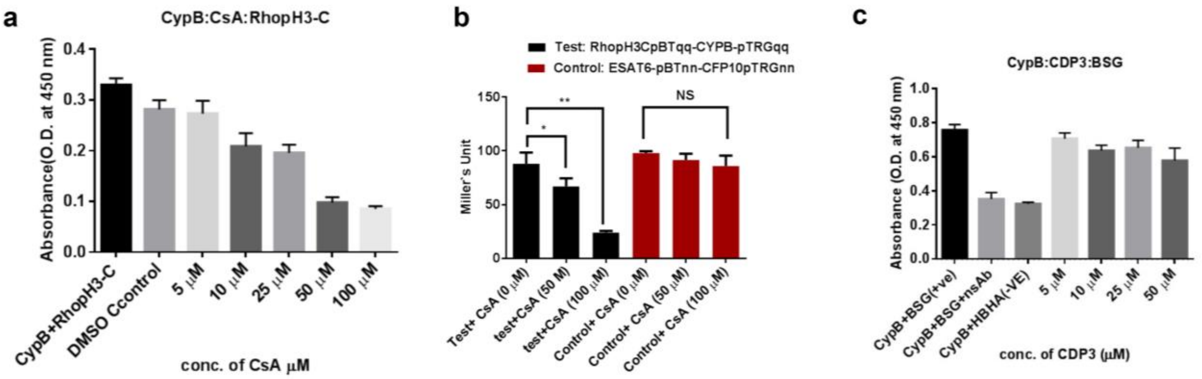

Supplementary figure 8. Disruption of interactions using CsA and CDP3.

(a) ELISA-based disruption of interaction between CypB and RhopH3-C. CsA was used to block the coated CypB on ELISA plate, followed by overlaying of RhopH3-C. CsA blocked the CypB-RhopH3-C intercation in a dose-dependent manner. Each bar represents the mean ± S.D. for triplicate experiment. **(b)** Liquid β-galactosidase assay using CsA as the inhibitor: CsA was added in liquid β-galactosidase assay culture containing RhopH3-CpBTqq/CYPBpTRGqq double cotranformants. ESAT6pBTnn/CFP10pTRGnn co-transformants were used as controls. The graph is the average of three independent assays with error bars representing the standard deviation. All values were tested for significance using a two-tailed unpaired Student's t-test with Welch`s correction. **P<0.01, NS:non-significant. **(c)** ELISA-based inhibition assay for CypB and Basigin interaction using CDP3. The CypB protein was coated on ELISA plate followed by CDP3 ovelaying. Subseqent to washing of unbound CDP3, BSG protein was ovelaid and binding was allowed for 1 h. To detect the binding, anti-Basigin (rabbit) antibody was added followed by anti-rabbit secondary antibody. Optical density was measured at 450 nm. ELISA-based inhibition assay showed that CDP3 is

not able to block the intercation between CypB and Basigin. Each bar represents the mean ± S.D. for triplicate experiment.

Supplementary Figure 9. (Prakash et al.)

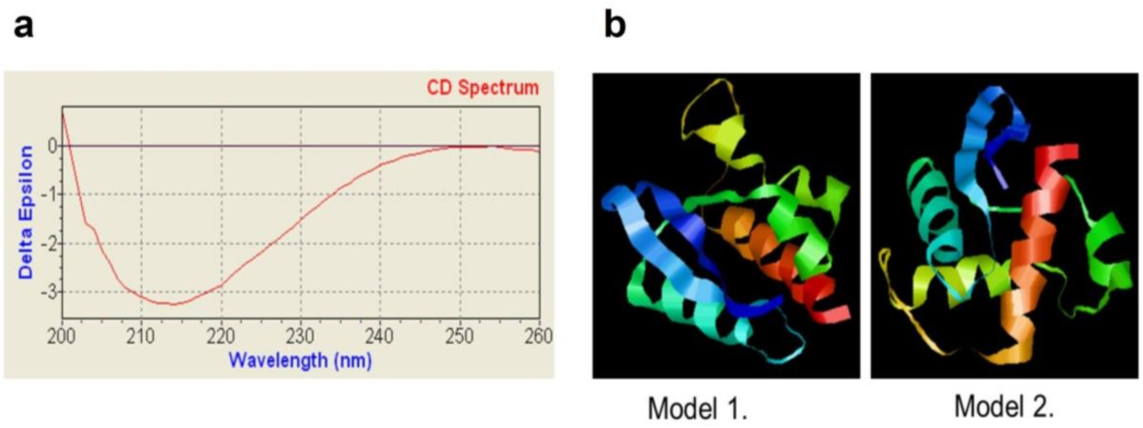

Supplementary Figure 9. (a) Circular Dichroism spectra of CDP3. The CD spectra for the protein at 0.2 mg/ml was analysed using the Circular Dichroism Neural Network (CDNN) and found to be composed of ~29% Helix, 19% β-sheets. **(b).** Quark server structure predictions (*ab initio* modeling from amino acid sequence http://zhanglab.ccmb.med.umich.edu/QUARK). Two conformations are represented as model 1 and 2. The predictions estimate ~40% α-helices and ~20% β-sheets.

Supplementary Figure 10. (Prakash et al.)

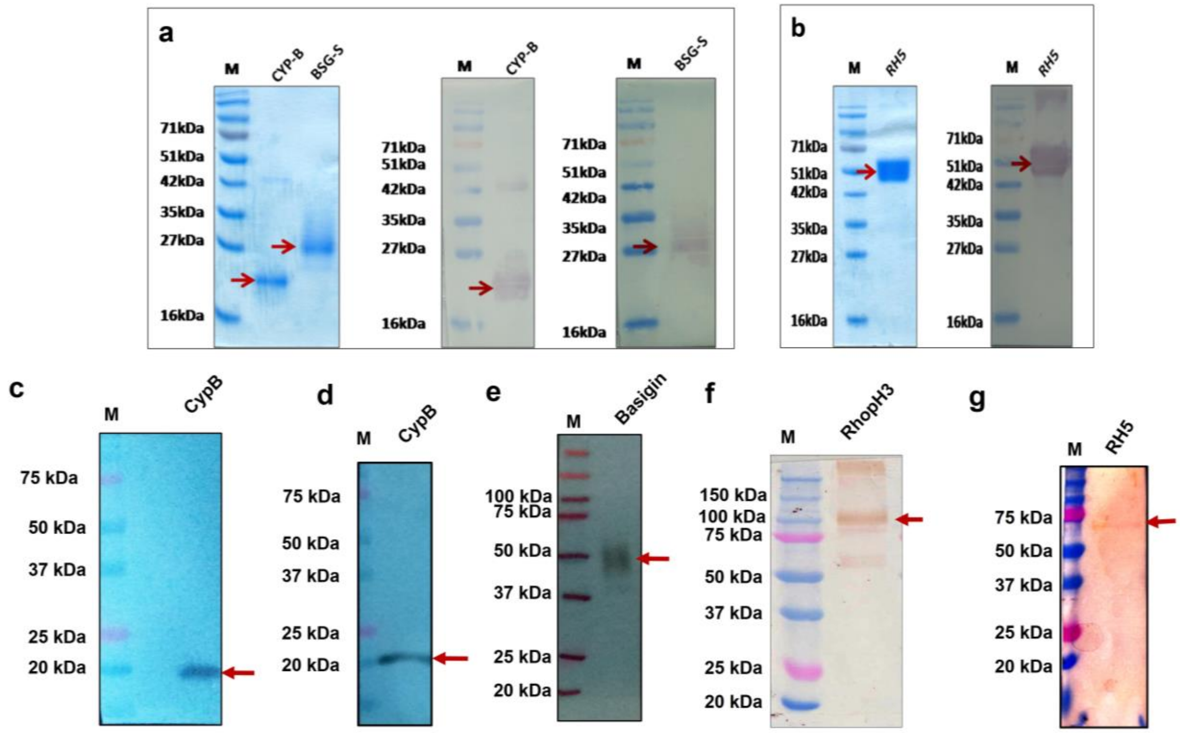

Supplementary Figure 10. Analysis of recombinant proteins and Native proteins. Recombinant proteins BSG, CypB and PfRh5 were analysed by SDS-PAGE and Western blotting: **(a)** SDS-PAGE and Western blot analysis of recombinant CypB and recombinant BSG. **(b)** SDS-PAGE and Western blot of PfRh5. **Western blot analysis of native proteins using their specific antibodies to check the quality of antibodies: (c)** CypB detection in RBC ghost using anti-CypB monoclonal (mouse) **(d)** anti-CypB polyclonal (rabbit) respectively. **(e)** anti-Basigin polyclonal (rabbit) used to detect native Basigin in RBC ghost. (**f)** Detection of native RhopH3 protein by anti-RhopH3-C polyclonal antibody (rabbit). **(g)** Detection of PfRh5 protein by anti-Rh5 (rabbit). (Schizont material was used to detect native PfRhopH3 and PfRh5).

Supplementary Figure 11. (Prakash et al.)

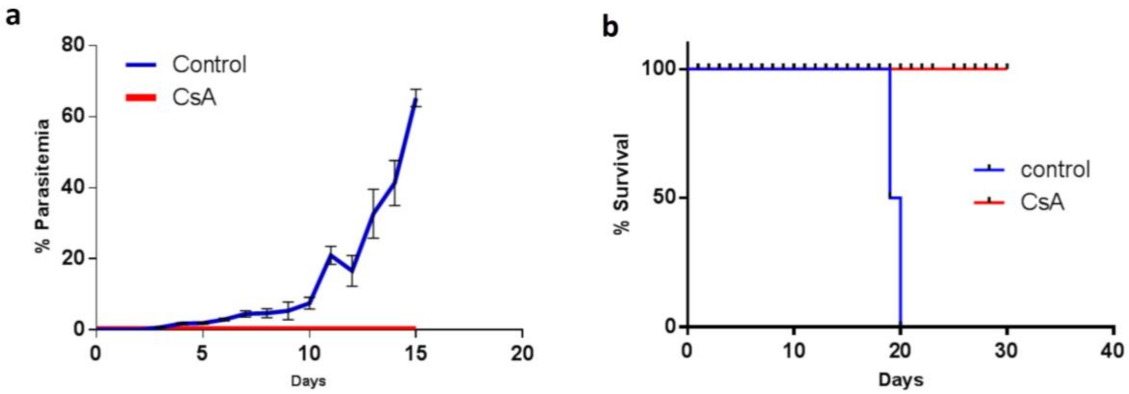

Supplementary Figure 11. *In vivo* effect of Cyclosporin A on parasitemia and survival. (a) Course of parasitemia in Balb/C mice treated with CsA (10 mg/kg per day) during *P. berghei* infection as compared with mice treated with buffer control. CsA treated mice show complete protection from parasite. **(b)** CsA treated mice showed 100% survival even up to 30 days of infection while in control 50% of mice died on day 19 and remaining 50% died the next day.

Supplementary Figure 12. (Prakash et al.)

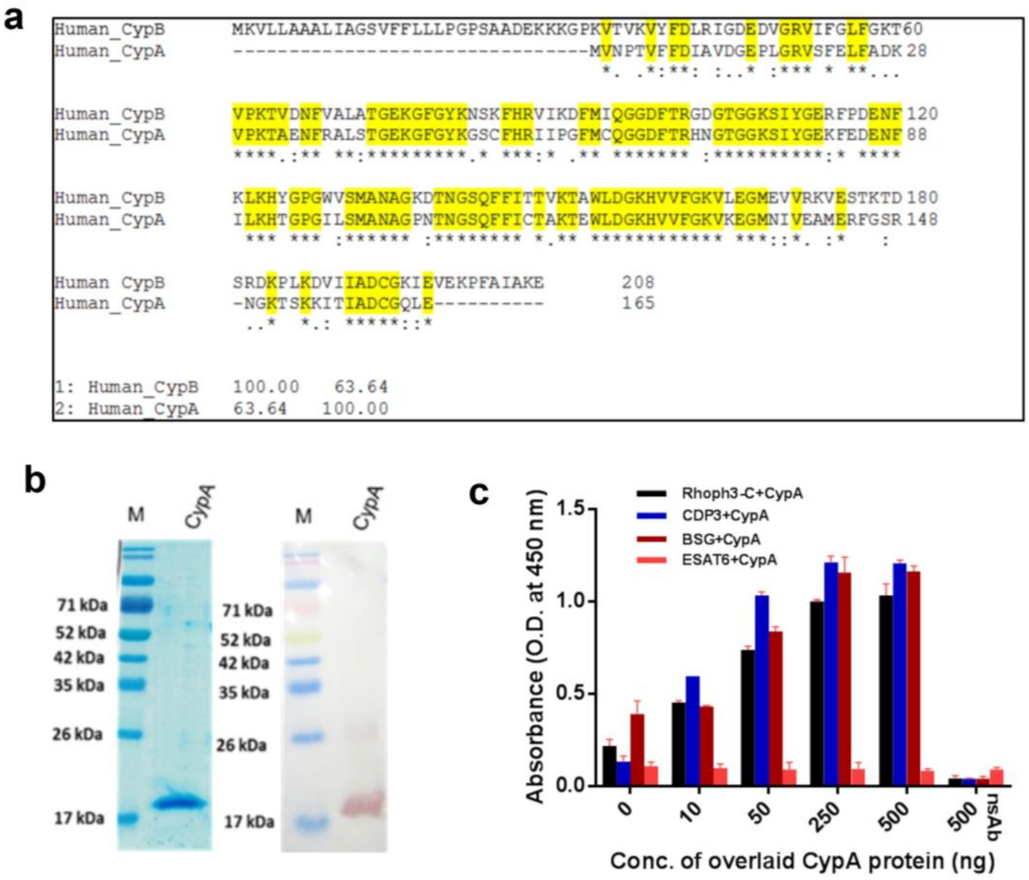

Supplementary Figure 12. Cyclophilin A interacts with RhopH3-C, CDP3 and Basigin. (a) Sequence alignment of CypA and CypB using Clustal-omega tool. **(b)** Analysis of the recombinant CypA protein by SDS-PAGE and Western blot using anti-CypA polyclonal antibody (rabbit). **(c)** ELISA-based interaction between CypA/RhopH3-C, CypA/CDP3 and CypA/Basigin. Mycobacterial ESAT6 protein used as negative control. Each bar represents the mean ± S.D. for triplicate experiment.

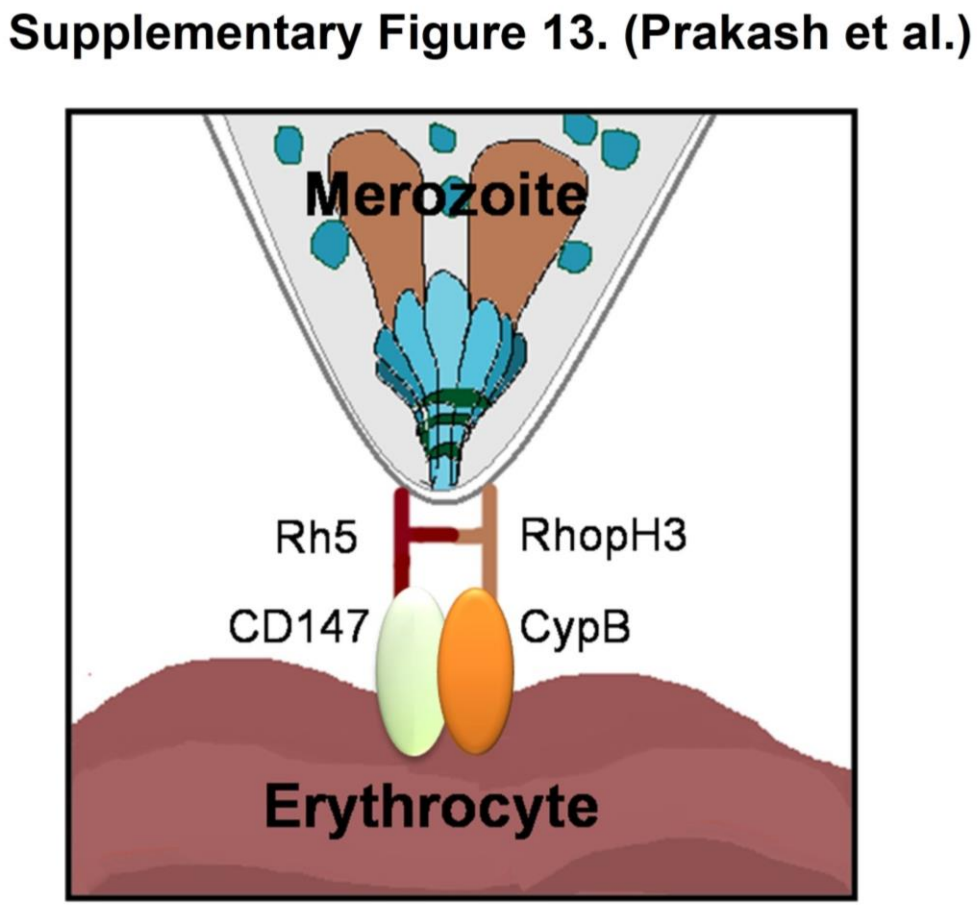

Supplementary Figure 13. Hypothetical Model explaining the multi-protein interactions. The merozoite proteins PfRhopH3 and PfRh5 interact with the RBC surface proteins Cyclophilin B and Basigin and aid the parasite invasion.

Supplementary Table 1.

CypB binding assay on merozoite:

S. No.	Binding	α-Rap2b	α-RhopH3-C
1	4000	1500	700
2	3600	4000	400
3	4000	4000	400
4	4000	4000	2000
5	4000	4000	4000
6	2000	4000	3600
7	3600	4000	2200
8	4000	4000	1150
9	3600	3800	2400
10	4000	4000	1700
**Mean**	**3680**	**3730**	**1855**

Intensity of CypB protein bound on the merozoite surface. Intensity highlighted with Red color is of those merozoites that are presented in Figure 2a. Intensity highlighted in blue color is of the merozoites shown in Supplementary Figure 4.

Supplementary Table 2.

Strains, Plasmids and Antibodies:

**Strains**	**Source/Reference(s)**
E. coli BL21 (DE3)	Novagen, USA
E. coli XL-1 Blue MRF::Kan^r	Stratagene, USA
E. coli R1 reporter strain	Stratagene, USA
P. falciparum 3D7	Malaria Research and Reference Reagent Resource Center (MR4) (Chloroquine Sensitive strain)
P. falciparum Dd2	MR4 (sialic acid dependent strain)
P. falciparum HB3	MR4 (sialic acid-independent strain)
P. falciparum 7G8	MR4 (chloroquine resistant strain)
**Plasmids**	
pTRG and pBT	Ref. [1]
pTRGnn and pBTnn	Ref. [1]
pMTSA	Ref. [2]
**Antibodies**	
Anti-His mAb HRP conjugated	Quiagen, Germany (34460)
Anti-CD147(Basigin) mAb (mouse)	Abcam, UK, [MEM-M6/1] (ab666)
Anti-CD147(Basigin) pAb (rabbit)	Santa Cruz Biotechnology, Inc. USA, (M-190): sc-25531
Anti-Cyclophilin B mAb (mouse)	Abcam, UK, [k2E2] (ab74173)

Anti-Cyclophilin B pAb (rabbit)	Abcam, UK (ab16045)
Anti-CFP10 pAb (rabbit)	Abcam, UK (ab45074)
Anti-PfRhopH3-C pAb (rabbit)	In-house
Anti-PfRhopH3-C pAb (mouse)	In house. Ref[3]
Anti-PfRh5 pAb (rabbit)	In-house. Ref[4]
Alexa Fluor 488 secondary antibody	Cell Signaling Technology, Inc. USA
Alexa Fluor 594 secondary antibody	Cell Signaling Technology, Inc. USA
IRDye 800CW-conjugated secondary antibody	LI-COR, Inc. USA

Supplementary Table 3.

Primer Sequences:

Primers	Sequence
Double stranded (ds) hairpin	5’TTTAAACACGTGGCGGCCGCTCTAGAGGCCCGC GCGGGCCTCTAGAGCGGCCGCCACGTGTTTAAA-3’
HP2P	P-5’-AGCGGCCGCCACGTGTTTAAA-3’
pBT For	5’-TGAGAGTTGTTCCGTTGTGGGGAA-3’
pBT Rev	5’-CCACAGGGTAGCCAGCAGCATCCT-3’
pTRG For	5’-CAACTGGAAGCTTTCGTTGACTTACGT-3’
pTRG Rev	5’-TAGAGGATCTCACTAGTTCATTAATTAATTA-3’
HP2PS1	5’CCGAATTCTACGTAAGCGGCCGCCACGTGTTTAAA-3’

Supplementary Table 4.

Screening of Human lung c-DNA library against RhopH3-C:

S.N.	Clones	Amino acid sequence	length
**1**	CypB	MKVLLAAALIAGSVFFLLLPGPSAADEKKKGPKVTVKVYFD LRIGDEDVGRVIFGLFGKTVPKTVDNFVALATGEKGFGYK NSKFHRVIKDFMIQGGDFTRGDGTGGKSIYGERFPDENFK LKHYGPGWVSMANAGKDTNGSQFFITTVKTAWLDGKHVV FGKVLEGMEVVRKVESTKTDSRDKPLKDVIIADCGKIEVEK PFAIAKE	208
**2**	HL1	NSDFYLLALIKI	12
**3**	HL3	AQFHSQETCDLCIIVIEDVSLMSVIL	26
**4**	HL4	AHFLCLSFGVYQKFGTRLISSVYLLGSIL	29

The Human lung c-DNA library (cloned in pTRG vector; library size 2.5 x 10^6, average insert size of 1.2 kb; http://www.biodynein.com/product-detail/2ad2c4d8480d01197adc355de49735ef) was screened to identify host interacting partners of PfRhopH3-C (cloned in pBT vector) using the bacterial two-hybrid assay. A total of 3 x 10^4 colonies were screened. Multiple blue colonies, indicative of protein-protein interaction, were obtained on the X-gal indicator plates. After colony re-plating, liquid patching, segregation, plasmid isolation, and finally sequencing of clones obtained from four prominently blue-coloured colonies, a single clone among them showed a sequence corresponding to the full-length human Cyclophilin B, while the other three clones showed frame-shifted cDNA inserts that did not correspond to any known human protein sequence. The CypB clone, therefore, was used as our protein of interest for the present study (listed in the table above).

Supplementary Table 5.

Screening of DIEL di-codon library against CypB:

S.N.	Clones	Amino acid sequence	length
1	CDP1	SGRHVFKSTDIDIDIGADIELDIDIDIFKHVAAAV	35
2	CDP2	SGRHVFKMHPGDINVDIMHSTMHQLMHGAMHPGMHELNV RNGAMHDIDIQLEINVDIFKHVAAA	64
3	CDP3	SGRHVFKMHDIDIDIDIDIDIGAMHWPELDIMHDIMHMHDIDIE LDISTDIMHELDIDIDIGADIMHMHDIDIDIGAELQLDIDIMHDIE LFKHVAAA	98
4	CDP4	SGRHVFKMHDIDIDIDIDIDIGAMHWPELDIMHDIMHMHDIDIE LDISTDIMHELDIDIDIGADIMHMHDIDIDIGAELQLDIDIMHDIE LFKHVAAA	98

The screening of our di-codon library (100% de novo proteins; library size 10^4) against CypB protein yielded 11 blue colonies on the X-gal indicator plate. We chose four of these colonies based on the intensity of their blue colour. Plasmids were isolated and segregated from each selected colony. Subsequently, R1 cells were co-transformed with the segregated plasmids and plated on X-gal indicator plates. Two clones were found to be of much lower intensity in their blue color (CDP1 and CDP2), while the blue-colour intensity of the other two clones was comparatively higher (CDP3 and CDP4). All these segregated plasmids were sequenced, of which CDP3 and CDP4 were found to possess the same sequence. A liquid-patch of these colonies is presented in Supplementary figure 7f.

Supplementary Table 6.

DNA sequence of PfRhopH3-C gene and CDP3 gene:

S. N.	Gene Name	Gene Sequence	Length
1	PfRhopH3-C (Codon-optimized)	**>RhopH3-C**<br>CTGTCCTGCTTTTATCCGAAGGAATTCGAACTGATTAAATCACGTAT<br>GATTCATCCGAACATCGTTGACCGCATTCTGAAAGGCATCGATAATC<br>TGATGAAGTCCACCCGTTATGATAAAATGCGCACGATGTACCTGGAT<br>TTTGAAAGCTCTGACATTTTCTCGCGTGAAAAAGTCTTTACCGCTCT<br>GTATAACTTTGATAGCTTCATCAAAACGAACGAACAGCTGAAAAAGA<br>AAAATCTGGAAGAAATCTCTGAAATCCCGGTCCAACTGGAAACCAGT<br>AATGATGGCATCGGTTATCGCAAGCAGGATGTGCTGTACGAAACCGA<br>CAAACCGCAAACGATGGATGAAGCGTCTTACGAAGAAACCGTGGATG<br>AAGACGCCCATCACGTTAACGAAAAACAGCATAGTGCACACTTCCTG<br>GACGCAATTGCTGAAAAAGATATCCTGGAAGAAAAGACCAAAGATCA<br>GGACCTGGAAATTGAACTGTATAAATACATGGGTCCGCTGAAGGAAC<br>AATCAAAATCGACCAGCGCGGCCTCTACGAGTGATGAAATCTCCGGC<br>TCAGAAGGTCCGTCTACCGAATCGACCAGCACGGGCAACCAGGGTGA<br>AGATAAGACCACGGACAATACCTATAAAGAAATGGAAGAACTGGAAG<br>AAGCAGAAGGCACGAGCAATCTGAAGAAAGGTCTGGAATTTTACAAG<br>AGTTCCCTGAAACTGGATCAACTGGACAAGGAAAAACCG	744
2	CDP3	**>CDP3**<br>AGCGGCCGCCACGTGTTTAAAATGCATGATATCGATATCGATATCGA<br>TATCGATATCGATATCGGCGCCATGCATTGGCCAGAGCTCGATATCA<br>TGCATGATATCATGCATATGCATGATATCGATATCGAGCTCGATATC<br>AGTACTGATATCATGCATGAGCTCGATATCGATATCGATATCGGCGC<br>CGATATCATGCATATGCATGATATCGATATCGATATCGGCGCCGAGC<br>TCCAGCTGGATATCGATATCATGCATGATATCGAACTCTTTAAACAC<br>GTGGCGGCCGCT	294

Supplementary Methods

Preparation of RhopH3-C antibody.

Two New Zealand white female rabbits were taken and immunized intramuscularly on day 0 with 150 µg RhopH3-C protein formulated with Freund's complete adjuvant (Sigma Chemical Company Co., USA) followed by three boosts emulsified with Freund's incomplete adjuvant on days 21, 42 and 63. Rabbits were bled on days 70 for sera collection. Antibody titres in serum sample was quantified by enzyme-linked Immunosorbent assay (ELISA).

Liquid β-galactosidase assay using Cyclosporin A as an inhibitor.

To show the inhibitory effect of CsA, we performed an *in vivo* experiment using the liquid β-galactosidase assay system[1]. For this, double co-transformant R1 cells: RhopH3-CpBTqq/CypBpTRGqq (test) and ESAT6-pBTnn/CFP10-pTRGnn (control) were inoculated overnight in LB media containing Chloremphenicol and Tetracycline. Secondary culture was inoculated with adequate antibiotics and 40 µM IPTG. At an O.D. ~0.6 at 600 nm, CsA was added to the culture at various concentrations (0 µM, 50 µM and 100 µM) and the culture incubated for a further 3 h. The assay was performed as mentioned in the methods section.

Binding and Localization of CDP3 on RBC surface

Uninfected human erythrocytes were incubated with 20 µM CDP3 protein followed by fixation with 4% paraformaldehyde and blocking with 10% FBS. Subsequently, cells were incubated with anti-His rabbit and anti-CypB mouse antibodies and probed with

anti-rabbit alexa-fluor 594 and anti-mouse alexa-fluor 488 secondary antibodies respectively. Cells were imaged with Nikon A1 confocal microscope.

Co-localization PfRhopH3 and CypB on merozoite surface.

Merozoites were incubated with 20 µM CypB protein followed by fixation with 4% paraformaldehyde and blocking with 10% FBS. Cells were then incubated with anti-RhopH3-C rabbit and anti-CypB mouse antibodies and probed with anti-rabbit alexa-fluor 594 and anti-mouse alexa-fluor 488 secondary antibodies respectively. Cells were imaged with Nikon A1 confocal microscope.

Co-localization of PfRHoph3-C and PfRh5 on merozoite surface.

Merozoites were fixed with 4% paraformaldehyde and subsequently blocked with 10% FBS. Merozoites were then incubated with anti-RhopH3 mouse and anti-Rh5 rabbit antibodies and subsequently probed with anti-rabbit alexa-fluor 594 and anti-mouse alexa-fluor 488 secondary antibodies. Cells were imaged with Nikon A1 confocal microscope.

ELISA-based inhibition of CypB/RhopH3-C interaction using Cyclosporin A.

The CypB protein was coated overnight on a 96-well ELISA plate (NuncMaxisorb ELISA plates) at 200 ng concentration, in 100 µl of 0.1 M carbonate/bicarbonate-coating buffer, pH 9.6. Coated wells were blocked using the blocking buffer (1xPBS, 0.1% Tween-20 and 3% BSA) for 1 h at 37 ºC, following which the plates were washed three times with washing buffer (1xPBST) and incubated for 1 hour at 37 ºC with different concentration of CsA (0 µM, 5 µM, 10 µM, 25 µM, 50 µM and 100 µM) in 100 µl of binding buffer (50 mM HEPES, 250 mM Potassium acetate and 5 mM Magnesium acetate, pH 8.0). Following washing (three times with the washing buffer), 400 ng/well of PfRHopH3-C

protein was overlaid in 100 µl binding buffer and incubated at 37 ºC for 1 h. Subsequent to three times washing with washing buffer, primary anti-RhopH3-C antibody (1:5000, rabbit) was added and the mixture incubated at 37 ºC for a further 1 h. After washing, secondary anti-rabbit antibody (HRP conjugated) was added (1:5000 dilution) and the plates incubated a further for 1 h. The plates were washed three times with washing buffer (1xPBST) and a further three times with 1xPBS, following which 100 µl of HRP substrate TMB was added and the plates incubated at 37 ºC for 30 min. Finally, Stop solution (1N H_2SO_4) was added and the optical density measured at 450 nm using an ELISA plate reader. DMSO was used as a negative control.

Supplementary References

1. Bhalla, K. *et al.* Host ICAMs play a role in cell invasion by *Mycobacterium tuberculosis* and *Plasmodium falciparum*. *Nature communications* **6**, 6049 (2015).

2. Tharad, M. *et al.* A three-hybrid system to probe in vivo protein-protein interactions: application to the essential proteins of the RD1 complex of *M. tuberculosis*. *PloS one* **6**, e27503 (2011).

3. Ranjan, R. *et al.* Proteome analysis reveals a large merozoite surface protein-1 associated complex on the *Plasmodium falciparum* merozoite surface. *Journal of proteome research* **10**, 680-691 (2011).

4. Chen, L. *et al.* Crystal structure of PfRh5, an essential *P. falciparum* ligand for invasion of human erythrocytes. *eLife* **3** (2014).
